# Immune response dynamics in COVID-19 patients to SARS-CoV-2 and other human coronaviruses

**DOI:** 10.1371/journal.pone.0254367

**Published:** 2021-07-09

**Authors:** Resmi Ravindran, Cindy McReynolds, Jun Yang, Bruce D. Hammock, Aamer Ikram, Amna Ali, Adnan Bashir, Tanzeel Zohra, W. L. William Chang, Dennis J. Hartigan-O’Connor, Hooman H. Rashidi, Imran H. Khan

**Affiliations:** 1 Department of Pathology and Laboratory Medicine, University of California, Davis, CA, United States of America; 2 Department of Entomology and Nematology, University of California, Davis, CA, United States of America; 3 National Institutes of Health, Rawalpindi, Pakistan; 4 California National Primate Research Center, University of California, Davis, CA, United States of America; 5 Department of Medical Microbiology and Immunology, School of Medicine, University of California, Davis, CA, United States of America; New York State Department of Health, UNITED STATES

## Abstract

COVID-19 serological test must have high sensitivity as well as specificity to rule out cross-reactivity with common coronaviruses (HCoVs). We have developed a quantitative multiplex test, measuring antibodies against spike (S) proteins of SARS-CoV-2, SARS-CoV, MERS-CoV, and common human coronavirus strains (229E, NL63, OC43, HKU1), and nucleocapsid (N) protein of SARS-CoV viruses. Receptor binding domain of S protein of SARS-CoV-2 (S-RBD), and N protein, demonstrated sensitivity (94% and 92.5%, respectively) in COVID-19 patients (n = 53), with 98% specificity in non-COVID-19 respiratory-disease (n = 98), and healthy-controls (n = 129). Anti S-RBD and N antibodies appeared five to ten days post-onset of symptoms, peaking at approximately four weeks. The appearance of IgG and IgM coincided while IgG subtypes, IgG1 and IgG3 appeared soon after the total IgG; IgG2 and IgG4 remained undetectable. Several inflammatory cytokines/chemokines were found to be elevated in many COVID-19 patients (e.g., Eotaxin, Gro-α, CXCL-10 (IP-10), RANTES (CCL5), IL-2Rα, MCP-1, and SCGF-b); CXCL-10 was elevated in all. In contrast to antibody titers, levels of CXCL-10 decreased with the improvement in patient health suggesting it as a candidate for disease resolution. Importantly, anti-N antibodies appear before S-RBD and differentiate between vaccinated and infected people—current vaccines (and several in the pipeline) are S protein-based.

## Introduction

Coronaviruses have long been recognized as important pathogens that cause both mild and severe respiratory diseases in humans [1, 2]. So far, seven HCoVs that can invade humans have been identified, including 229E, NL63, HKU1, OC43, SARS-CoV, MERS-CoV, and SARS-CoV-2, causing the present pandemic [1, 3, 4]. NL63 and 229E are α-coronaviruses and OC43 and HKU1 are β-coronaviruses that frequently infect and cause a mild common cold-like illness [5]. Antibodies to these common cold HCoVs are present in most individuals (>90%) [6]. SARS-CoV and SARS-CoV-2 and MERS-CoV are zoonotic β-coronaviruses that have recently crossed into humans and caused severe illness [5]. Even with antivirals available, the key to controlling the spread and preventing more virulent strains is rapid detection and isolation of suspect cases. Serological methods for detecting SARS-CoV-2 infection will enable the evaluation of several important aspects in the fight against COVID-19: sub-clinical disease missed by molecular diagnostic methods, testing contacts of COVID-19 positive individuals, proof of previous infection, making links between clusters to help epidemiological investigations, assessment of COVID-19 vaccine efficacy, and prevalence information to develop effective measures to lower the rate of new infections [7–9].

Although molecular diagnostic tests have been developed to detect SARS-CoV-2 infection, serological assays are important as a complement to PCR in the diagnosis of infection. PCR tests can detect SARS-CoV-2 during the period of viral shedding but the duration of viral shedding is not well understood [10]. Importantly, RT-PCR may yield up to 29 percent false-negative results globally; 10 percent in the USA [11].

It has been reported that COVID-19 patients seroconvert and develop antibodies against SARS-CoV-2 antigens after 6 to 21 days of clinical symptoms [4]. Hence serological tests have generated substantial interest as an alternative and/or complement to RT-PCR. However, serological tests may have high false-positive results due to cross-reactivity with common coronaviruses. Therefore, carefully developed and validated serologic assays are critical for patient contact tracing, identifying the viral reservoir hosts, and epidemiologic studies [7]. Serologic assays are also needed for the evaluation of results of vaccine trials and monitoring therapeutic antibodies.

A key limitation of conventional serological assays is that they can detect only a single coronavirus antigen in each serum sample resulting in an inefficient testing system. A substantial number of the new commercial COVID-19 antibody serological tests are lateral flow assays, which provide a simple positive or negative result, with no quantitative information and lack the desired sensitivity and specificity [8]. To circumvent this limitation, well-characterized multiplexed serological assays are needed. We have previously demonstrated the diagnostic validity of a bead-based multi-analyte serological assay in tuberculosis (TB) based on testing for plasma antibodies to specific *Mycobacterium tuberculosis* antigens [12–18]. We have successfully implemented a multiplex antibody assay for 17 mouse pathogens including a mouse coronavirus for routine sentinel screening at the Comparative Pathology Laboratory (University of California Davis) and Jackson Laboratories (Bar Harbor, Maine, USA) [19, 20].

Here, we report the development and validation of a multiplex bead assay to simultaneously and specifically detect antibodies to the SARS-CoV-2, SARS-CoV, MERS-CoV, and common human coronavirus strains (229E, NL63, OC43, HKU1). Furthermore, multiplex serological assays enabled the investigation of the dynamics of immune responses (antibodies including immunoglobulin subtypes, and cytokines/chemokines) in COVID-19 patients.

## Materials and methods

### Ethics statement

Plasma from patients and healthy individuals were obtained under the protocols approved through the relevant Institutional Review Boards (IRBs) at the University of California, Davis Medical Center, National Institute of Health (NIH), Pakistan as well as School of Biological Sciences (SBS), University of the Punjab, Lahore, Pakistan. Written informed consent was received from all participants before inclusion in the study and all the samples were de-identified before access. All COVID-19 de-identified samples were obtained according to protocols approved by the IRB and Pathology & Laboratory Medicine’s Clinical Research Oversight Committee at the University of California, Davis Medical Center, as well as IRB at the NIH, Pakistan (IRB:1584225, IRB 218204).

Animals (non-human primates, and rabbits) were housed according to the Guide for the Care and Use of Laboratory Animals and the standards outlined by the American Association for Accreditation of Laboratory Animal Care; all animal experiments were performed under approval from Institutional Animal Care and Use Committees at the UCDavis, Rutgers University, NJ as well as Rocky Mountain Laboratories (National Institutes of Health (NIH)) [18, 21, 22].

### Recombinant proteins

The following were produced under Federal contract HHSN272201400008C and obtained through Biodefense and Emerging Infections Research (BEI) Resources Repository, NIAID, NIH: Spike (S) glycoprotein receptor binding domain (S-RBD) from SARS-CoV-2 (Wuhan-Hu-1 with C-Terminal Histidine Tag recombinant protein expressed in HEK293F Cells; NR-52366); SARS-CoV Spike Protein deltaTM recombinant expressed in Baculovirus (NR-722); and SARS-CoV Nucleocapsid (N) recombinant protein expressed in *Escherichia coli* (NR-48761). MERS-CoV Spike protein fragment (RBD, aa367-606, His Tag) (40071-V08B1); 229E Spike Protein (S1 Subunit, His Tag) (40601-V08H); NL63 Spike Protein (S1 Subunit, His Tag) 40600-V08H; HKU1spike glycoprotein Protein (aa 1–760, His Tag) 40021-V08H; and OC43 Spike Protein (S1+S2 ECD, His Tag) 40607-V08B, were obtained from Sino Biological Inc. (Wayne, PA).

### Animal models of SARS-CoV-2 infection

Rhesus macaque (*Macaca mulatta*) serum used as a positive control was courtesy of Emmie de Wit, Ph.D. (emmie.dewit@nih.gov) through BEI Resources: Pooled nonhuman primate (NHP) convalescent serum raised against SARS-CoV-2, Gamma-Irradiated, (NR-52401) were collected after 21 days post-infection from four adult animals inoculated with SARS-CoV-2 (isolate nCoV-WA1-2020). Serum samples were confirmed for neutralization and reactivity in ELISA. In addition, plasma samples (pre-and day 12 post-inoculation) were obtained from a rhesus macaque experimentally infected with SARS-CoV-2, isolated from the nasal swab of a COVID-19 patient admitted to the University of California Davis Medical Center (UCDMC), Sacramento (courtesy of Dr. Smita Iyer, Center for Immunology & Infectious Diseases, UCDavis) [21]. Plasma samples were also obtained from seven rhesus macaques immunized with adenovirus vaccine for SARS-CoV-2 S protein collected at 6 weeks post-boost. Samples from 103 healthy, seronegative macaques were used in the study. These were archived plasma samples collected before 2019 and cryopreserved at -80C [14]. The first group consists of rhesus macaques (n = 64) obtained from a specific pathogen-free (SPF) colony at the CNPRC at UC Davis. Plasma samples from the second group consisted of healthy Rhesus macaques (n = 39) at the AAALAC-accredited SPF colony at the Charles River Laboratories (Wilmington, MA).

### COVID-19 patient plasma samples (UCDMC, BEI resources and NIH-Pakistan)

All COVID-19 patients were confirmed by RT-PCR testing for SARS-CoV-2. The date of illness onset, clinical classification, RT-PCR testing results during the hospitalization period, and personal demographic information was obtained from the clinical records. Samples were drawn, processed, and stored under a standardized protocol: centrifuged immediately after the blood draw, plasma aliquoted and stored at -80°C.

#### UCDMC

Samples were obtained from hospitalized patients (n  =  6) with symptomatic illness and confirmed for SARS-CoV-2 infection by RT-PCR. These plasma samples were anonymized, collected upon hospital admission and submitted to the UC Davis Comprehensive Cancer Center shared resources Biorepository, aliquoted, and stored at -80°C until use. The median age of the patients was 62 years (IQR, 54–69 years). From each patient, longitudinal samples collected for a period of several days to weeks were obtained. These samples were collected between one- and 37-days post-symptom onset (as reported by patients). The list of the SARS-CoV-2 patient samples from UCDavis included in the study with basic demographic and clinical information can be found in S1 Table.

#### COVID19 specimens from BEI resources

Four COVID confirmed human plasma samples were obtained from Bei resources. The demographic and clinical information of these patients is not available.

#### COVID19 specimens from Pakistan

Forty-three COVID PCR-confirmed human plasma samples were obtained from the National Institutes of Health (NIH), Pakistan; anonymized samples, collected under the institutional ethical approval protocol at NIH, Pakistan. The median age of the patients was 59 years (IQR, 52–69 years), and 28% were females. Basic demographic and clinical information of the SARS-CoV-2 patient from Pakistan included in the study can be found in S2 Table.

### Healthy control group

These samples served as negative controls in this study; collected before SARS-CoV-2 epidemic and cryopreserved at −80°C. This group comprised of archived pre-pandemic EDTA plasma samples of mixed-sex collected from California Central Valley (Delta Blood Bank (Stockton, CA)), (n = 75) [23]. Healthy controls were between the ages of 18 to 85 years (median age 51 years; IQR: 39, 60) who self-reported to have no respiratory or other infections. Also, pre-pandemic EDTA blood samples of healthy individuals (n = 54) of mixed sex (median age 21 years; IQR: 20, 23) were taken from Pakistan; these individuals had no history of pulmonary symptoms, and no known medical conditions (infection, cancer, or metabolic disease) [16]. This group consisted of random, young individuals to represent the general healthy population for comparison to COVID-19 patients.

### Respiratory disease control patient samples (other than COVID-19)

#### Animal models

The following serum samples were obtained through BEI Resources:

Rabbit Sera Control Panels (NR-4569): NR-4569 consists of two control panels containing pooled polyclonal sera obtained from rabbits dosed with a recombinant truncated form of the SARS-CoV spike protein in the absence (NRC-768) or presence (NRC773) of adjuvant—serum samples from individual rabbits with low, medium and high virus neutralization titers were pooled to prepare the panels. Each panel also consisted of pooled sera from rabbits dosed with PBS. Sera from healthy rabbits (n = 9) were courtesy of Dr. Selvakumar Subbian, Rutgers University, NJ.

#### Human patients

Convalescent serum 001 (NR-18964) and 002 (NR-18965) to 2009 H1N1 influenza A virus (n = 2) and reference antiserum to the respiratory syncytial virus (NR-32832) (n = 4) were obtained from BEI Resources. NR-32832 is a panel of sera containing: 1) pooled reference antiserum to the respiratory syncytial virus (RSV)1, 2) three pooled control antiserum preparations of varying titers to RSV, 3) reference immune globulin to RSV, prepared from pooled human plasma, and 4) serum depleted of immunoglobulin G (IgG) for use as a negative control.

The pre-pandemic negative control panel was complemented by a panel of plasma samples collected between 2017 and 2018 from individuals (n = 66) with chronic obstructive pulmonary disease (COPD) from Pakistan (median age of 60 years; IQR: 52, 65) [16]. Archived plasma samples from TB patients (n = 32) from Pakistan (median age 38 years; IQR: 29, 51) from a previously published retrospective study were also used as a disease control group [16]

### Coupling of HCoV antigens to Luminex microbeads

Recombinant viral antigens for microbead coating were obtained from BEI Resources or Sino Biological Inc. (Wayne, PA) (Described in detail in the section Recombinant Proteins under Materials and Methods) Carboxylated microbeads were purchased from Luminex Corp. (Austin, TX). Various antigen preparations were covalently conjugated to the microbeads as previously described [24]. Briefly, an aliquot of 2.5 ×10^6^ beads was removed and resuspended in 80 μl of activation buffer (100 mM monobasic sodium phosphate; pH 6.2) by vortexing and sonication. To activate the beads for cross-linking to proteins, 10 μl of 50-mg/ml sulfo-N-hydroxysulfosuccinamide (Pierce, Rockford, IL) and 1-ethyl-3-[3-dimethylaminopropylcarbodiimide (EDC; Pierce, Rockford, IL). The bead mixture was shaken on a rotary shaker at room temperature for 20 min and washed twice with 250 μl phosphate-buffered saline (PBS), pH 7.4. The beads were resuspended in the relevant antigen preparation diluted in PBS buffer and incubated by mixing on a rotator for 2 h at room temperature. Beads were washed twice with 250 μl PBS, resuspended in 250 μl of blocking buffer (1% BSA; 0.1% Tween 20 in PBS, pH 7.4; 0.05% sodium azide), and mixed on a rotator at room temperature for 30 min. After blocking, beads were resuspended in 1 ml of blocking buffer and stored at 2–8°C in dark. The optimal concentration for each antigen was determined by coupling different microbead sets with 6.25ug/ml and 25μg/ml for each HCoV antigen. Bead sets were also coated with bovine serum albumin (BSA, 100 μg/ml) as a negative control protein (Pierce, Rockford, IL) and goat anti-Human IgG (20ug/ml) as a positive control (Bethyl, TX).

### Multiplex microbead immunoassay

Multiplex assays were performed based on the xMAP platform (Luminex Corp, Austin, TX) and data (median fluorescence intensity (MFI) were collected as previously described [15, 16]. Two separate assays were used to evaluate the IgG and IgM response to antigens from SARS-CoVs, MERS, and common cold HCoVs. In brief, a mixture of microbead sets, one for each of the coated antigens described above, were incubated with the participants’ plasma specimens, which were diluted 1:200 in 2% Prionex (bio-WORLD, Dublin, OH) for 1 hour at room temperature in a 96-well plate. After incubation, the beads were washed twice by adding 100 μl of wash buffer (PBS-tween) per well and drained under vacuum using a vacuum manifold (Millipore Corporation, Bedford, MA). For detection of human and NHP IgG, phycoerythrin-conjugated anti-human IgG was used (Jackson ImmunoResearch, Pennsylvania) at a 1:500 dilution in PBS-tween, and incubated at room temperature for 15 min. Following incubation, beads were washed two times with wash buffer, resuspended in 100 μl of wash buffer per well, and analyzed in the Magpix instrument.

For detection of human IgM, biotinylated anti-human IgM was used (BD Biosciences Cat# 555781) at a 1:500 dilution and for the detection of rabbit IgG, biotinylated anti-rabbit IgG (Vector Laboratories, Burlingame, CA) at a 1:1000 dilution in 2% Prionex was used, and the assay was performed as described previously [18].

### IgG isotyping

To identify the antigen-specific antibody per IgG subclasses, biotinylated IgG1 (Southern Biotech Cat# 9052–08) was added at 5ug/ml and biotinylated IgG2, IgG3, or IgG4, specific detection reagents (Southern Biotech Cat#s 9052–08, 9060–08, 9210–08, 9200–08) were added at 20 ug/ml in 2% Prionex and a customized Luminex subclassing assay was used as described previously [18].

### Multiplex cytokine/chemokine assay

Multiplex kits for measuring cytokines, chemokines, and growth factors (Cat#12007283), for use on the Luminex platform (Luminex Corp., Austin, TX), were obtained from Bio-Rad, Hercules, CA. Assays were performed according to the manufacturer’s instructions. There were 48 immune molecules/analytes (cytokines/chemokines) in the assay kit that included: FGF basic, Eotaxin, G-CSF, GM-CSF, IFN-γ, IL-1β, IL-1ra, IL-1α, IL-2Rα, IL-3, IL-12 (p40), IL-16, IL-2, IL-4, IL-5, IL-6, IL-7, IL-8, IL-9, GRO-α, HGF, IFN-α2, LIF, MCP-3, IL-10, IL-12 (p70), IL-13, IL-15, IL-17A, CXCL-10 (IP-10), MCP-1 (MCAF), MIG, β-NGF, SCF, SCGF-β, SDF-1α, MIP-1α, MIP-1β, PDGF-BB, RANTES, TNF-α, VEGF, CTACK, MIF, TRAIL, IL-18, M-CSF, TNF-β. The concentration (pg/ml) of each cytokine/chemokine in the multiplex panels was measured based on a 7-point standard curve using xPONENT 4.3 software (Luminex, TX).

We also developed an in-house assay for the quantitation of CXCL-10 in human plasma samples and validated using the Luminex platform. CXCL-10 capture and detection antibodies and purified protein were purchased from R&D systems, MN. Optimal parameters for quantitation were first determined, including capture and detection antibody concentrations, buffers, and matrix effects. Briefly, beads coupled to 200ug/ml capture antibody (Cat# MAB266) were incubated with plasma samples diluted 1:4 in 1% BSA/PBS for 1 hour at room temperature. A standard curve was generated using recombinant human CXCL-10 Protein (Cat# 266-IP) ranging from 10,376 pg/mL to 3 pg/mL. The beads were washed two times by adding 100 μl of PBS-tween per well and draining under vacuum. Biotinylated anti-CXCL-10 (Cat# BAF266) was used at 0.8ug/ml in 1% BSA/PBS and incubated at room temperature for 1 hour. Beads were washed two times and 50 μl of streptavidin-phycoerythrin (SA-PE) (CalTag, Burlingame, CA) was added at a dilution of 1:500 in wash buffer. The assay was incubated at room temperature for 15 min, beads were washed two times with wash buffer, resuspended in 100 μl of wash buffer per well, and analyzed in the Magpix instrument. The concentration (pg/ml) of CXCL-10 was measured based on a 7-point standard curve using xPONENT 4.3 software (Luminex, TX). The sensitivity and specificity of the in-house assay were compared to CXCL-10 multiplex kit from Biorad (Hercules, CA) and the correlation coefficient R^2^ was 0.82. We used this in-house assay to measure the CXCL-10 concentrations in COVID-19 patients from Pakistan.

### Development of a quantitative assay for SARS-CoV-2 IgG titer determination

To create a standard curve for the SARS-CoV-2 S-RBD assay, a COVID-19 patient plasma sample with the highest MFI was used as a standard. This standard plasma was serially diluted and tested by the multiplex assay. The MFI values and dilutions were plotted using 5-parameter logistic (5PL) regression analysis by the use of GraphPad Prism Version 9.0.0 (GraphPad Software Inc., La Jolla, California, http://www.graphpad.com/scientific-software/prism). The highest plasma dilution (1/12,800) with the lowest antibody level was set to titer 1, and the lowest was set to 100. The interpolated value for each unknown COVID-19 positive sample was obtained by 5PL regression analysis.

### Data analysis

For the analysis of antibody data, cut-off values were calculated for each antigen-coated microbead set using data from healthy individuals for Indian and Pakistani samples separately (Cutoff = Mean MFI + (3 standard deviations). Separate cut-off values were determined for each secondary reagent (IgG, IgM, IgG1,2,3,4). The cut-off values were used to determine antibody positive samples in the data sets from the US and Pakistan.

For measurements of antibodies, and cytokines/chemokines, graphs were generated, and p-values were determined by one-way ANOVA and Tukey’s multiple comparison test using GraphPad Prism. To compare differences between multiple groups, data were tested with 1-way ANOVA and Tukey’s multiple comparison test and adjusted P values were reported.

### Animal housing

NHPs were maintained in cages with 4 square feet of floor space, or 6 square feet if over 10 kg, with fixed perch bars in a temperature-controlled vivarium with continuous monitoring of temperature and humidity. All animals had visual and auditory access to other macaques 24 hours per day and were fed a balanced commercial macaque chow (Purina Mills, Gray Summit, MO) twice daily with fresh produce twice weekly, and free access to water 24 hours per day. Supplemental food was provided when clinically indicated. Environmental enrichment was provided daily, including manipulanda (forage boards, mirrors, puzzle feeders) and novel foodstuffs. Veterinarians, animal health technicians, and staff technicians conducted daily health/clinical assessments of animals. For vaccinations and blood collections, animals were anesthetized by intramuscular injection (i.m) of ketamine-HCl (Parke-Davis, Morris Plains, NJ) at 10 mg/kg of body weight. For virus inoculation and nasal secretion sample collection, animals were additionally anesthetized with 15–30 ug/kg dexmedetomidine HCl inject i.m. and anesthesia was reversed with 0.07–0.15 mg/kg atipamezole HCl injected i.m. Analgesics were given to minimize pain and discomfort at the discretion of the veterinary staff and nutritional supplements were administered, as necessary. When euthanasia was necessary, animals were humanely euthanized at the end of the study by a barbiturate overdose and necropsy procedures were performed by veterinary pathologists and support staff. Criteria for assessments of health and well-being of the animals were as follows: no signs of injury, distress or pain that cannot be alleviated by analgesics, weight loss, hypothermia, persistent anemia, chronic dehydration, lethargy, severe dyspnea, neurological deficits, coagulopathies, motor retardation etc. If an animal’s physical condition deteriorated prior to the scheduled endpoint, clinical veterinary staff would have euthanized the animal following the Guidelines for Humane Euthanasia of Animals on Projects (GHEAP) at the CNPRC. In this study, none of the macaques were euthanized for welfare reasons before completion. Animals infected with SARS-CoV-2 were euthanized at the end of the study and animals in the vaccine group were returned to their home colony since the study was not terminal.

## Results

### Multiplex antibody profiling in nonhuman primate (NHP) and rabbit models

**Fig 1:**
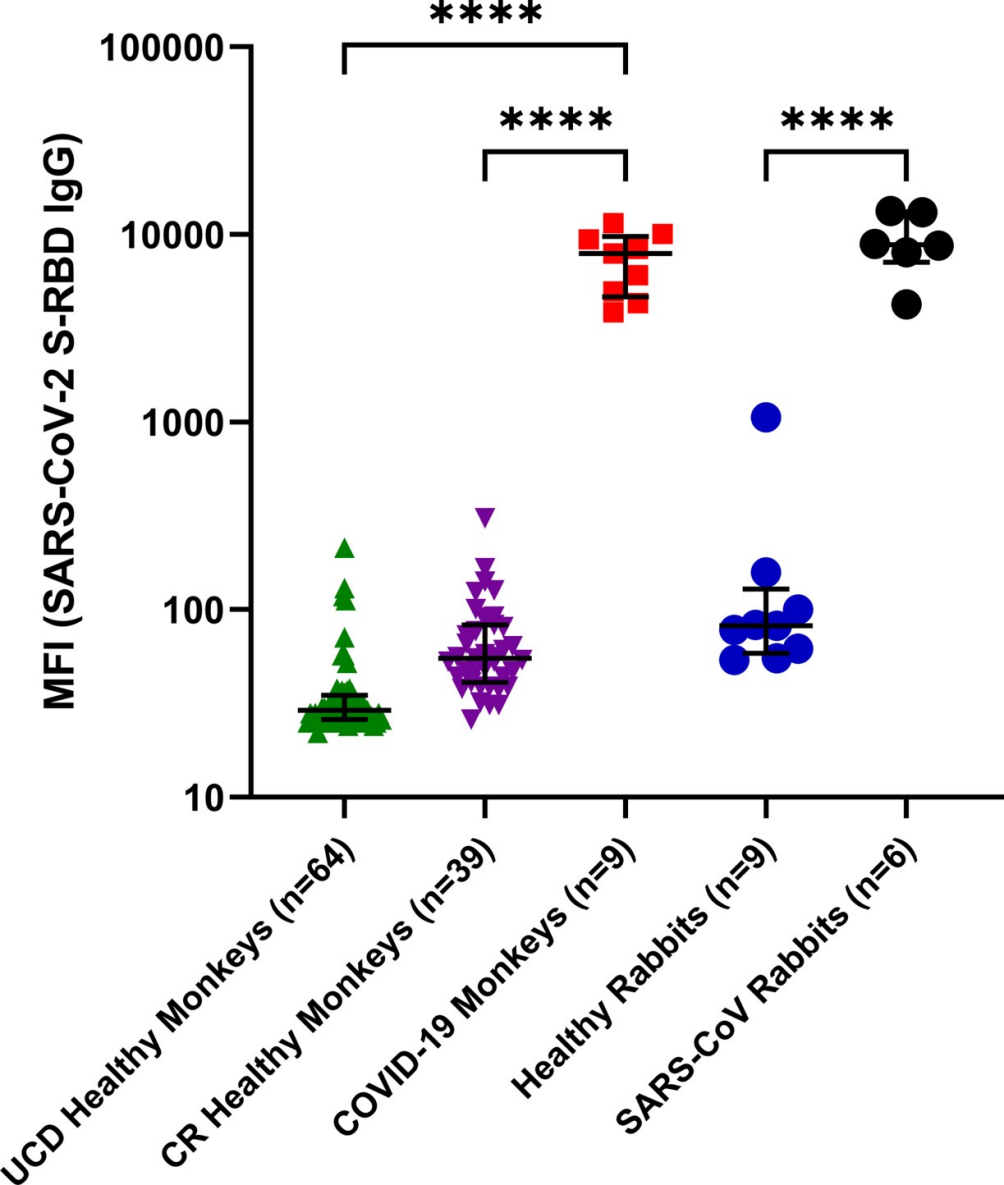
Detection of specific antibody responses (IgG) against SARS-CoV-2 S Protein RBD domain (S-RBD) in rhesus macaques and rabbits as detected by the multiplex assay. Multiplex assay was performed to detect IgG antibodies against SARS-CoV-2 S-RBD, SARS-CoV S & N, MERS-CoV S-RBD and S proteins of 4 common coronaviruses. IgG antibodies only to SARS-CoV-2 (S-RBD) antigen as determined in a multiplex microbead assay format are shown as MFI here. MFI values in log10 scale with Interquartile Range (IQR) are shown. Healthy monkeys from UCD (UC Davis) and CR (Charles River), and rabbits were included as negative control animals. Statistical significance was tested by one-way ANOVA and Tukey’s multiple comparison test (P value <0·0001 is denoted by ****).

The multiplex antibody assay was tested using NHP sera from infected or vaccinated monkeys (n = 9). One sample was pooled sera from a group of rhesus macaques in the convalescent phase following SARS-CoV-2 infection (BEI: NR-52401). Other samples included an animal infected with SARS-CoV-2 (UCDMC isolate), and seven NHPs vaccinated using adenoviral vectors expressing the SARS-CoV-2 S-RBD protein. Strong antibodies (IgG) to SARS-CoV-2 S-RBD were detected in all nine (Fig 1). In one animal (NHP) infected with SARS-CoV-2 obtained from CNPRC, SARS-CoV-2 S-RBD specific antibodies (IgG) were detected at 12 days post-infection. In addition, we detected SARS-CoV-2 S-RBD specific antibodies (IgG) six weeks after boosting vaccination (Fig 1). Healthy NHP controls at CNPRC and Charles River Laboratories did not contain antibodies to S-RBD (Fig 1).

In the rabbit model, six animals immunized with SARS-CoV S protein reacted to both the SARS-CoV-2 S-RBD (Fig 1) and SARS-CoV S (S3 Table) antigens in our multiplex antibody assay. This is expected because RBD in the S Proteins of the two viruses contains 74% amino acid identity [25].

### Sensitivity and specificity of antisera against SARS-CoV-2 S-RBD

**Fig 2:**
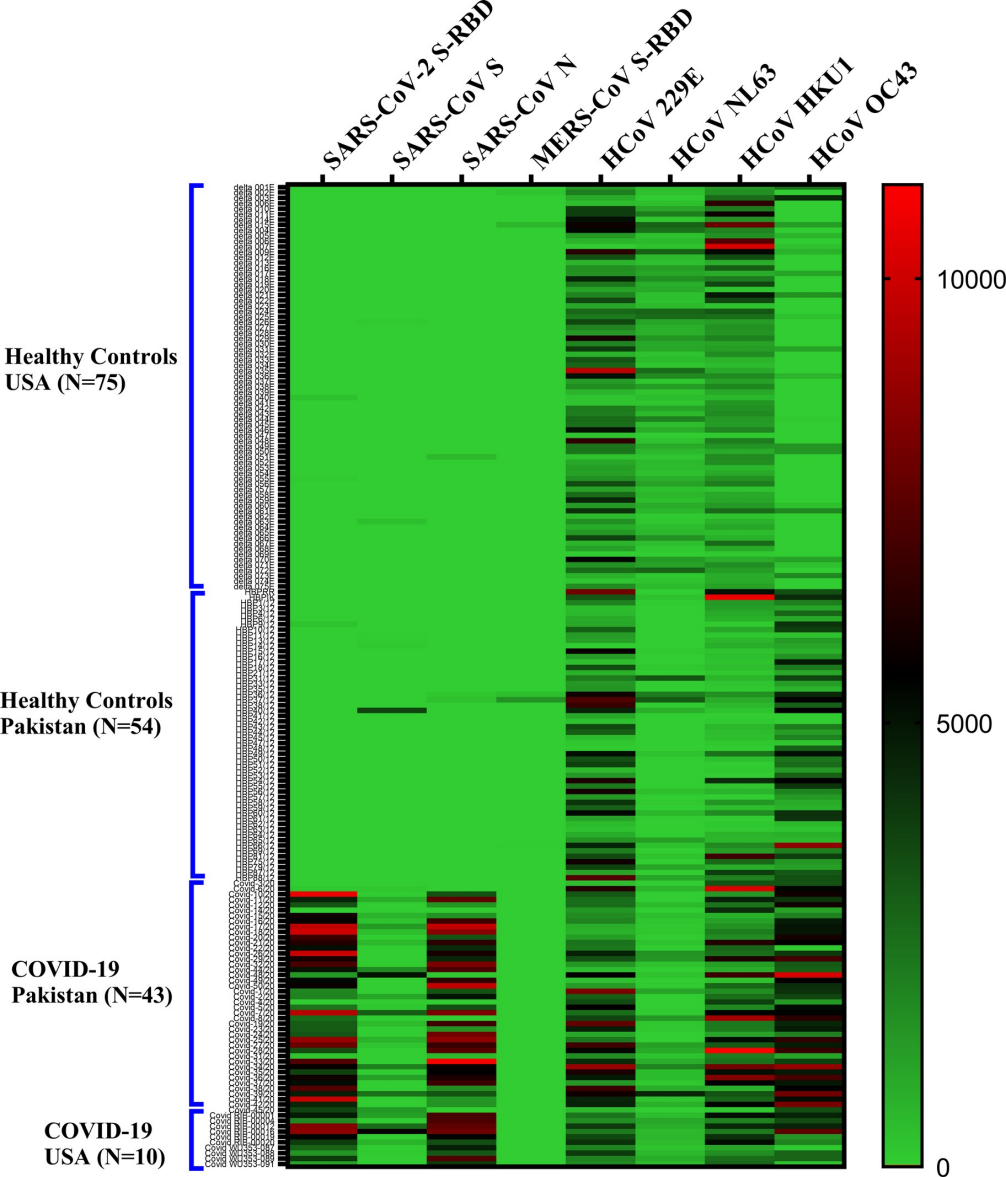
Heat map depicting overall antibody responses detected by multiplex microbead panel against members of the coronavirus family (SARS-CoV-2; SARS-CoV; MERS-CoV and 4 common coronaviruses (229E, NL63, OC43, HKU1)) in COVID-19 patients and healthy controls. Antibody responses (IgG) to SARS-CoV-2 S-RBD; SARS-CoV S and N; MERS-CoV S-RBD; and S proteins of 4 common coronaviruses are shown. In patients where multiple time points were available, the latest time point after the onset of symptoms is shown. Each row corresponds to one sample and columns correspond to CoV antigens in the multiplex assay. The color intensity scale represents the relative MFI (median fluorescent intensity) values ranging from the highest (10,000; red) to no antibody response (0; green). No significant background reactivity to SARS-CoV-2 S-RBD, SARS-CoV S, SARS-CoV N, and MER S-RBD was detected in healthy controls from US and Pakistan.

Antibodies detected by multiplex antibody assay for seven members of the human coronavirus family are shown in a heatmap (Fig 2). Positive control plasma samples from confirmed COVID-19 patients (BEI resources; n = 4) were positive for antibodies against S-RBD of SARS-CoV-2 with some cross-reactivity to S protein of SARS-CoV (as well as to S proteins from common coronaviruses). Plasma samples from confirmed COVID-19 patients (n = 49) were similarly positive (clinical details in S1 and S2 Tables). All COVID-19 samples cross-reacted to N protein of SARS-CoV. This is likely due to the fact N proteins of SARS-CoV-2 and SARS-CoV share 90% amino acid identity [26] Healthy controls from US and Pakistan did not exhibit significant background reactivity to SARS-CoV-2 S-RBD, SARS-CoV S, SARS-CoV N, and MER S-RBD. Antibodies against the 4 common coronaviruses were present in majority of the healthy individuals.

**Fig 3:**
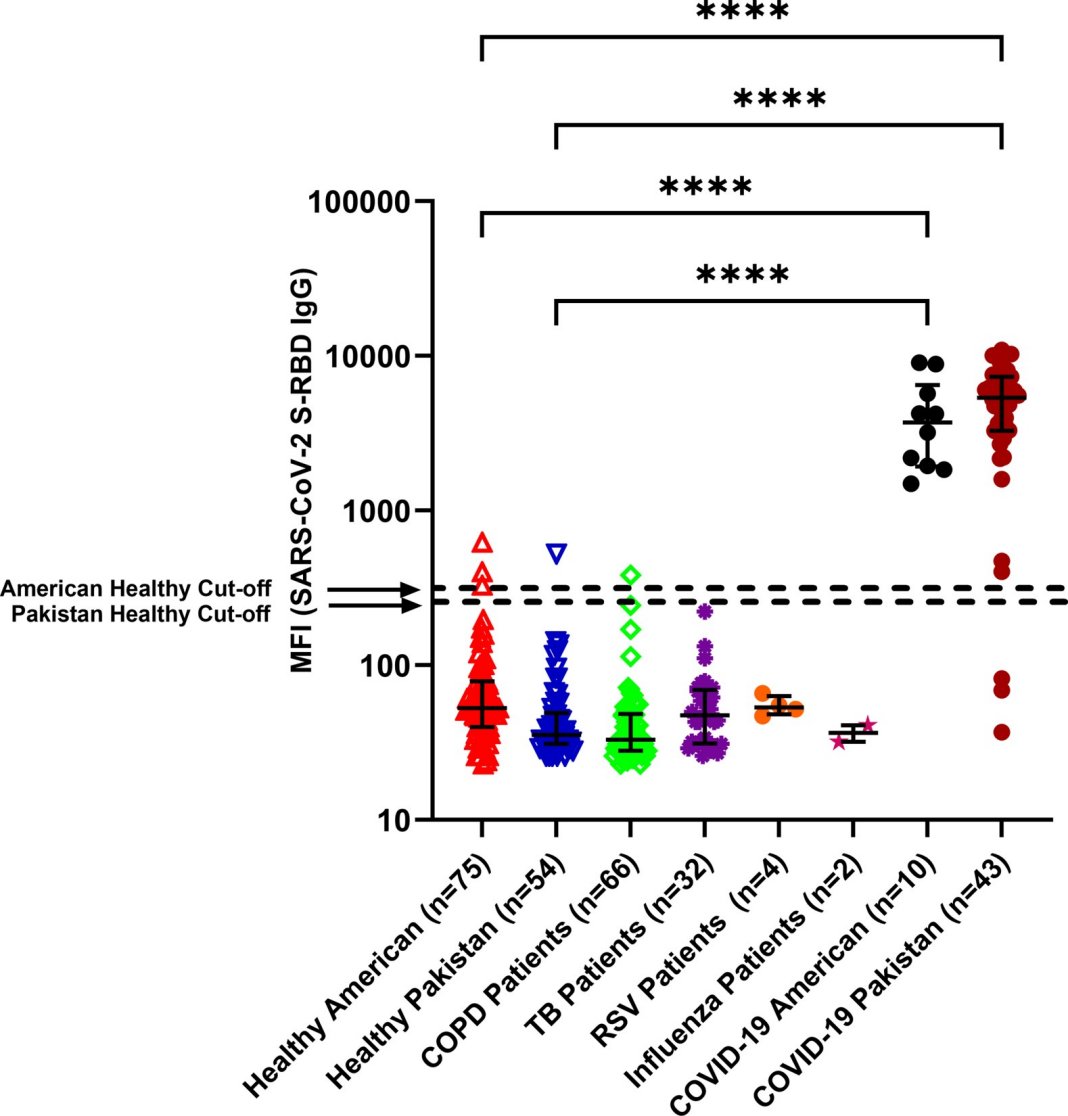
Antibodies (IgG) against SARS-CoV-2 S-RBD protein in COVID-19 patients and negative controls as detected by the multiplex assay. Multiplex assay was performed to detect antibodies against SARS-CoV-2 S-RBD, SARS-CoV S & N, MERS-CoV S-RBD and S proteins of 4 common coronaviruses. IgG antibodies to SARS-CoV-2 (S-RBD) antigen as determined in a multiplex microbead assay format are shown as MFI here.—MFI values in log10 scale with IQR are shown. Assay cutoff values for SARS-CoV-2 S-RBD IgG are indicated by dashed lines. Samples from pre-pandemic healthy individuals and patients with other respiratory diseases (COPD, TB, RSV, and influenza) were compared to COVID-19 patients from the USA and Pakistan. In patients where multiple time points were available, the latest time point after the onset of symptoms is shown. Statistical significance was tested by one-way ANOVA and Tukey’s multiple comparison test (P value <0·0001 is denoted by ****).

Among subjects with COVID-19, the antibody profiles against the seven viruses in the coronavirus family members were shown to be similar in the USA (n = 10) and Pakistan, n = 43). Healthy controls from both countries were devoid of antibodies against SARS-CoV-2 (USA, n = 75: Pakistan, n = 54) (Fig 3).

To assess whether other human respiratory viruses stimulated antibodies that cross-reacted with the recombinant SARS-CoV-2 S-RBD, we tested plasma samples from subjects with COPD (n = 66, Pakistan), tuberculosis (n = 32, Pakistan), influenza A (n = 2, USA), or respiratory syncytial virus infections (n = 4, USA) (Fig 3). All of these known disease control samples were devoid of antibodies to SARS-CoV-2 (S-RBD). For the negative control groups, the majority of the samples had MFI values below the cutoff for SARS-CoV antigens. The number of false-positive samples in healthy individuals from both USA and Pakistan were 3 out of 129 (2/75 in USA and 1/54 in Pakistan) samples tested for SARS-CoV-2 (S-RBD) and the assay specificity was 97.7%. The levels of antibodies in plasma samples from COVID-19 patients were significantly higher compared to healthy controls in both USA and Pakistan (p<0·0001).

To evaluate the specificity of the recombinant SARS-CoV-2 S-RBD, samples collected from different populations (USA and Pakistan combined) before the COVID-19 pandemic (n = 129; Fig 3, leftmost two columns) were used as negative control plasma. None contained antibodies against SARS-CoV-2 S-RBD; however, a majority contained antibodies against the four common coronaviruses (Fig 2). Levels of antibodies against OC43 strain appeared to be higher in healthy controls from Pakistan compared to healthy controls from the USA.

### Antibodies (IgG) against SARS-CoV-2 S-RBD, SARS-CoV S and N, MERS-CoV S-RBD, and common HCoVs S detected by multiplex antibody assay

**Fig 4:**
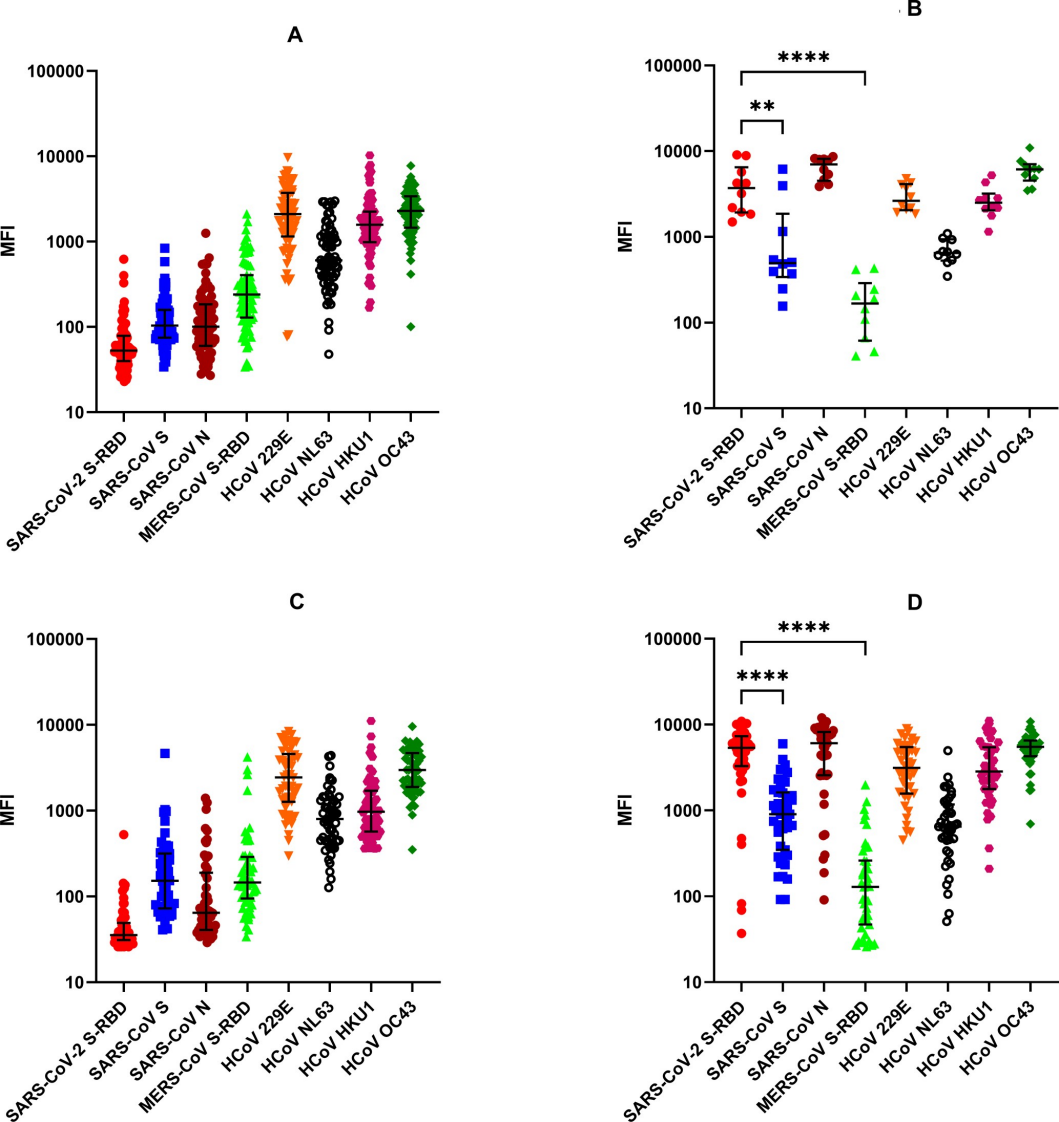
Total IgG levels detected by the multiplex antibody test against SARS-CoV-2 S-RBD, SARS-CoV S & N, MERS-CoV S-RBD and HCoVs. MFI values in log10 scale and IQR are shown, and the P values by one-way ANOVA and Tukey’s multiple comparison test between the groups are as follows: ** p<0.001, **** p<0.0001. (A) Healthy Controls from USA, (B) COVID-19 Patients from USA, (C) Healthy Controls from Pakistan, and (D) COVID-19 Patients from Pakistan. In patients where multiple time points were available, the latest time point post-onset of symptoms is shown.

Plasma samples collected from healthy adults in the USA (n = 75) before the SARS-CoV-2 pandemic contained high levels of antibodies to the recombinant S proteins of 229E, OC43, NL63, and HKU1 CoVs but not to SARS-CoVs (Fig 4A). In contrast, high levels of antibodies to SARS-CoV-2 were detected in all ten COVID-19 patients from USA; in cases where multiple time points are available, the latest time point post-symptoms is shown (Fig 4B). All ten samples cross-reacted to N protein of SARS-CoV. The two viruses, SARS-CoV and SARS-CoV-2, contain 90% amino acid identity in N protein [26]. To a lesser degree, there was cross-reactivity of samples from COVID-19 patients to SARS-CoV S protein; the two S proteins and S-RBDs share 76% and 74% amino acid identity, respectively [25]. In COVID-19 patients, levels of SARS-CoV-2 IgG antibodies were significantly higher compared to SARS-CoV S and MERS IgG antibodies (p<0·01 and p<0·05 respectively) (Fig 4B). Levels of SARS-CoV-2 S-RBD IgG antibodies were similar in comparison to SARS-CoV N (not significant, p>0·05) (Fig 4B). Antibodies against the 4 common coronaviruses in COVID-19 patients were similar to the healthy controls (Fig 4B).

We also tested archived pre–SARS-CoV-2 pandemic plasma samples collected from healthy individuals in Pakistan (n = 54) (Fig 4C). As in the case of healthy adults from the United States, most of the individuals from Pakistan had high levels of antibodies to the S proteins of common HCoVs but no antibodies to the RBD of SARS-CoVs were detected (Fig 4C). Only one of the 54 pre-pandemic healthy controls from Pakistan had low levels of SARS-CoV-2 S–RBD reactive IgG antibodies above the assay cut-off (98% specificity). Anti-SARS-CoV S and MERS S-RBD IgG was also detected in six healthy controls.

Antibody reactivity in COVID-19 patients from Pakistan was similar to that in patients from the USA (Fig 4D). All the plasma samples also cross-reacted strongly with the SARS-CoV N protein, but they were less reactive with the SARS-CoV S. Significantly higher levels of SARS-CoV-2 S-RBD IgG antibodies compared to SARS-CoV S were detected (p<0·0001) while the antibodies to SARS-CoV N was not significantly different. 93% of the patients were positive to SARS CoV-2 S-RBD protein (40/43) while three patients did not have antibodies to CoV-2 S-RBD protein.

### Antibodies (IgG) against SARS-CoV-2 S-RBD in COVID-19 patients with varying disease severity

We assessed IgG responses to SARS-CoV-2 S-RBD in the 43 COVID-19 patients from Pakistan stratified by disease severity. The levels of antibodies in plasma samples in severe COVID-19 patients (ICU, required intubation, n = 20) were higher (Median MFI = 5983) than in to mild to moderate COVID-19 patients (Median MFI = 3994; n = 23); however, the results were no significant (p-value = 0.2631).

### Anti-nucleocapsid (N) protein antibodies

This multiplex assay also detects antibodies against N proteins. All the COVID-19 plasma samples from USA and Pakistan cross-reacted strongly with the SARS-CoV N protein (Fig 4B and 4D). The two viruses, SARS-CoV and SARS-CoV-2, contain 90% amino acid identity in N protein [26]. Patterns of SARS-CoV N antibodies were identical to those of S-RBD of SARS-CoV-2 (Fig 4B and 4D). Three COVID-19 patients from Pakistan who did not have antibodies to SARS-CoV-2 S-RBD were also devoid of antibodies against SARS-CoV N protein. We speculate the RT-PCR results for SARS-CoV-2 infection may have been false positive; false positivity of RT-PCR can be as high as 16.7% [27].

In individuals who received the COVID-19 vaccine (Moderna, n = 10, and Pfizer, n = 7) antibodies against SARS-CoV-2 S-RBD were detected in all the subjects after 2 to 3 weeks of the first dose increasing exponentially after 2 weeks of the second dose, while none of the subjects contained antibodies to SARS-CoV N protein (manuscript under preparation; these authors).

### Kinetics of seroconversion and development of antibody subtypes

**Fig 5:**
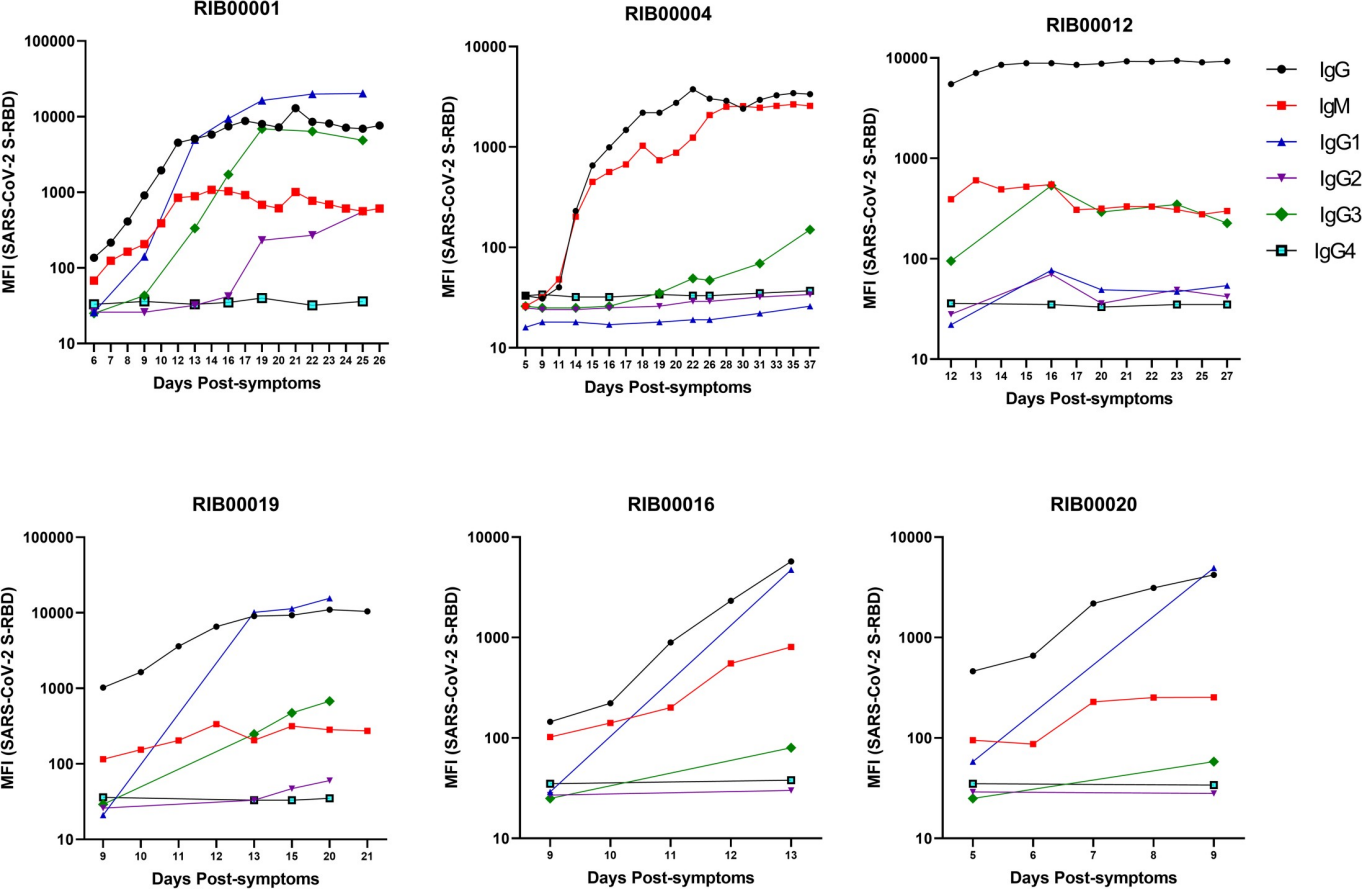
Kinetics of development of antibody subtypes (IgG, IgM, and IgG subtypes-1, 2, 3 and 4) in COVID-19 patients against SARS-CoV-2 S-RBD. Multiplex assays were performed individually to detect each antibody subtype against SARS-CoV-2 S-RBD, SARS-CoV S & N, MERS-CoV S-RBD, and S proteins of 4 common coronaviruses. Humoral immune responses against SARS-CoV-2 measured as MFI (log10) in longitudinal samples collected from six COVID-19 patients are shown at estimated time (days) from the onset of symptoms (as reported by patients) is shown.

We measured IgG, IgM, and IgG subtype (IgG1, IgG2, IgG3, IgG4) responses to S-RBD in plasma samples from six COVID-19 patients at several time points post-symptoms in separate assays (Fig 5). IgG and IgM antibodies appeared 5 days post-symptoms in two patients (RIB00012 and RIB00020). Between 10 to 14 days after appearance of symptoms, all 6 patients developed high levels of IgG and IgM antibodies against S-RBD as measured by the multiplex antibody assay. Patients reached a plateau after 2 weeks, except RIB00016 and RIB00020. Our results indicate that COVID-19 patients seroconvert between 5 to 10 days post- symptoms. Thus, S-RBD of SARS-CoV-2 is a highly sensitive and specific antigen for the detection of antibodies within a few days post-symptoms.

Among the IgG subtypes, IgG1 and IgG3 antibodies were detected in 3 patients (RIB00001, RIB00019, and RIB00016) at approximately 10–13 days post-symptoms. RIB00012 contained only IgG3 whereas RIB00020 contained IgG1 antibodies 9 days post-symptoms. In RIB00004, low levels of IgG3 antibodies were detected at approximately 37 days post-symptoms. IgG2 antibodies were detected in only one patient (RIB00001) at approximately 19 days post-symptoms. None of the other 5 patients contained IgG2 or IgG4 antibodies. We also measured the IgG subtypes in COVID-19 patients from Pakistan. IgG1 was present in 60% (12/20) of the severe COVID-19 patients and 57% (13/23) of mild to moderate COVID-19 patients (S4 Table). IgG3 antibodies against SARS-CoV-2 S-RBD were detected in 40% (8/20) of the severe COVID-19 and 13% (3/23) of mild to moderate COVID-19 patients. Interestingly, 65% (13/20) of the severe COVID-19 and 43% (10/23) of mild to moderate COVID-19 patients contained IgG3 antibodies against SARS-CoV N protein. IgG2 antibodies were present in one patient and no patient contained IgG4 antibodies (S4 Table).

### Plasma cytokines/chemokines in COVID-19 patients

**Fig 6:**
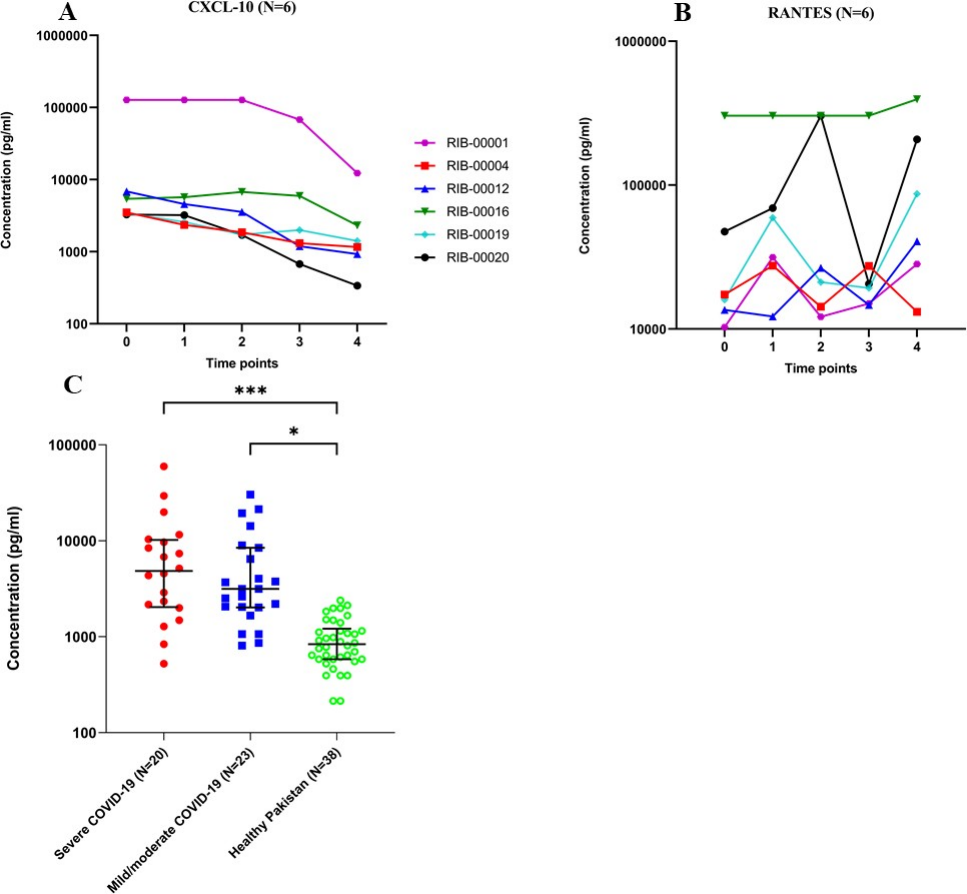
Dynamics of cytokines/chemokines in COVID-19 patients. Plasma concentrations (pg/mL) of (A) CXCL-10 and (B) RANTES in six COVID-19 patients from USA were detected by multiplex microbead assay. Estimated time (8–16 days) from the onset of symptoms (as reported by patients) is shown; (C) CXC-L10 in 43 COVID-19 patients from Pakistan. Concentration (pg/ml) and Interquartile Range (IQR) are shown, and the P values by one-way ANOVA and Tukey’s multiple comparison test between the groups are as follows: * p<0.05, ***p<0.001.

A multiplex panel of 48 analytes was used to assess longitudinal samples collected from six COVID-19 patients. Levels of inflammatory cytokines/chemokines, including Eotaxin, G-CSF, Gro-α, CXCL-10, RANTES (CCL5), IL-2Rα, MCP-1, and SCGF-b, were found to be highly elevated in plasma samples from COVID-19 patients within a week post-symptoms as compared to healthy controls (S5 Table). Notably, the most prominent chemokine CXCL-10 dropped precipitously in 6 of 6 patients over time as symptoms improved; all patients recovered (Fig 6A). By contrast, RANTES levels substantially increased with time in five of six patients (Fig 6B).

We also compared CXCL-10 levels in the 43 COVID-19 patients from Pakistan stratified by disease severity using an in-house assay developed by us (Fig 6C). Healthy controls (n = 38) were compared to severe (n = 20) and mild to moderate COVID-19 patients (n = 23). CXCL-10 levels were significantly elevated in both severe and mild/moderate COVID-19 cases compared to healthy controls (*** p<0·001 and * p<0.05 respectively). The median CXCL-10 concentration in severe and mild to moderate COVID-19 patients compared to healthy individuals was 7 and 3.8-fold higher, respectively. The median concentration of CXCL-10 in plasma samples in severe COVID-19 patients was 2-fold higher than mild to moderate COVID-19 patients, however, the difference was not statistically significant (p-value = 0.3931) (Fig 6C).

### Assay quantitation

Relative quantitation of antibody responses (IgG) to S-RBD (SARS-CoV-2) was determined based on a standard curve (MFI values plotted against serial dilutions of a standard plasma sample) (S1 Fig). In all six patients, the titers increased rapidly to peak in 5 to 15 days post-symptoms (S2 Fig). Strong correlations of antibody titers and MFI values of samples (R^2^) at various stages of the disease, at several time points, in each patient were demonstrated as follows (S3 Fig): RIB00001 (R^2^ = 0.89); RIB00004 (R^2^ = 0.98), RIB00012 (R^2^ = 0.99), RIB00019 (R^2^ = 0.97), RIB00016 (R^2^ = 0.82), and RIB00020 (R^2^ = 0.96).

## Discussion

Detection of blood-based immune biomarkers is generally practical and minimally invasive for health monitoring. We have developed a novel multiplex immunoassay to detect IgM and IgG antibodies to SARS-CoV-2 with high sensitivity (94%) and specificity (98%) (≥10 days post-symptoms). This multiplex panel, in addition, profiles antibodies against six other coronaviruses (SARS-CoV, MERS, and four common coronaviruses). The assay can be used to complement current detection platforms for SARS-CoV-2 infection and provide information regarding several baseline serological parameters such as infection with other coronaviruses and their impact on COVID-19, course of maturation of antibody responses in infection, optimal antigen selection for vaccine development, and vaccine efficacy, including the degree of cross-reactivity with emerging strains.

We compared the MFI values for antibodies against individual antigens generated by single-plex assays and multiplex assay for HCoV S protein antigen to investigate the possible change in MFI values when the antigens are multiplexed. We found no change in MFI between single-plex and multiplex assay formats.

### Animal models

The multiplex assay was validated using samples from animal models: rabbits and nonhuman primates. In NHP, experimentally infected with SARS-CoV-2 or vaccinated with vectored SARS-CoV-2 S-RBD, sensitivity was 100%, with 98% specificity (Fig 1). In rabbits, antisera developed against SARS-CoV S protein were found to be cross-reactive with SARS-CoV-2 S-RBD in the multiplex assay, as expected (Fig 1) [28]. Identical results were obtained in experimentally infected Syrian Golden hamsters manuscript under preparation; these authors.

### Human subjects

Our assay was validated using well-characterized human plasma samples. None of the healthy individuals or patients suffering from respiratory diseases other than COVID-19 contained antibodies to the S-RBD of SARS-CoV-2 (p<0·0001) (Fig 3).

Limited cross-reactivity with the SARS-CoV S protein and strong cross-reactivity with SARS-CoV N protein for IgG in COVID-19 patients was observed. These results are expected because the two viruses are closely related [25]. N-protein of SARS CoV-2 and SARS-CoV share 90% amino acid identity [26].

Our assay differentiates between vaccinated and non-vaccinated individuals by measuring antibodies against the N protein; the current vaccines, and several in the pipeline, are all based on the S protein (in various formats). Our current multiplex assay contains N antigen of SARS-CoV with 92.5% sensitivity and 98% specificity; inclusion of N protein of SARS-CoV-2 is in progress. None of our study subjects containing antibodies against the S proteins of common HCoVs contained antibodies against N protein of SARS-CoV, demonstrating there is no cross-reactivity with common coronaviruses.

SARS-CoV S-RBD and full-length S protein contain 74% and 76% amino acid identity, respectively [25]. Antibodies to SARS-CoV-2 S were cross-reactive to S protein of SARS-CoV but not MERS S-RBD. S protein of MERS shares only 34% amino acid identity with that of SARS-CoV-2 which likely explains the lack of cross-reactivity [29].

As expected, healthy controls contained antibodies against the 4 common coronaviruses, and similarly, so did COVID-19 patients [30]. HCoV reactive antibodies were present in a majority of adults while SARS-CoV-2 S-RBD cross-reactivity was rare (3 of 129; 2.3%). In pre-pandemic healthy controls from the USA (n = 75) and Pakistan (n = 54), low levels of IgG cross-reactive antibodies against SARS-CoV-2 S-RBD were detected in two and one plasma sample, respectively. Antibodies against SARS-CoV S Protein and MERS S-RBD were detected in six healthy individuals in Pakistan. We speculate that these are not cross-reactive antibodies against SARS-CoV-2 S-RBD and these healthy individuals may contain antibodies to SARS CoV and MERS. This may be because of the proximity of Pakistan to the Middle East where the MERS outbreak started [31].

The above results show that a majority of healthy individuals are devoid of cross-reactive antibodies to the recombinant proteins of SARS-CoVs. However, in contrast to our findings, a recent study reported antibody cross-reactivity between seasonal HCoVs and SARS-CoV-2 [30]. The second study comparing pre-pandemic blood samples from the United States, Tanzania, and Zambia identified antibodies against the 4 common coronaviruses that also reacted to SARS-CoV-2 and showed the prevalence of SARS-CoV-2 serological cross-reactivity was significantly higher in samples from sub-Saharan Africa compared to that in the USA [32].

Cross-recognition of SARS-CoV-2 by pre-existing memory helper T cells against common cold coronaviruses in individuals who had never been exposed to SARS-CoV-2 has been reported [33]. Common coronaviruses such as the NL63 RBD exhibit 20% sequence identity and HKU1 harbors a 2% sequence identity with the SARS-CoV-2 RBD [34]. However, the hypothesis of cross-reactive immunity between SARS-CoV-2 and common cold HCoVs still awaits experimental trials.

We measured the IgG and IgM antibodies in six COVID-19 patients where plasma samples were available at several time points over many weeks. Our results indicate that these patients seroconvert between 5 to 10 days post-symptoms. We also studied the dynamics of IgG subtype development in six patients where plasma samples were available at several time points over many weeks. We found elevated IgG3 antibodies in 5 out of 6 patients and IgG1 in 4 out of 6 patients as early as 9 to 13 days post-symptoms. Our results, although derived from a small group of subjects, are in line with previous findings by Amanat and colleagues [35]. In one patient IgG2 antibodies emerged after 19 days post-symptoms. None of the patients contained IgG4 antibodies which is anticipated given that this subtype develops largely in nonviral diseases [36].

Our assay is relatively quantitative and measures titers of SARS-CoV-2 S-RBD IgG antibodies (S2 Fig). The results also show a strong correlation between MFI values and sample dilution series (S3 Fig), showing either relative antibody titers or direct MFI values, can be used for antibody quantitation. This flexibility is afforded by the fact that MFI values as reported by the Luminex platform have a high dynamic range; from 0 to 20,000 MFIs. Unlike standard ELISA (limited to upper end of optical density of 2.0) a majority of samples with high levels of antibodies do not require dilution to fall within an acceptable dynamic range (S1 Fig).

Longitudinal analysis was performed to monitor the dynamics of cytokine and chemokine production in six COVID-19 patients where plasma samples were available over several time points post-symptoms. Samples were taken at the time of hospital admission (5 to 9 days post-symptoms; Time 0) and over several days/weeks. Plasma levels of CXCL-10 decreased over time compared to Time 0, (Fig 6A). Cytokines/chemokines are fragile molecules and their concentrations in blood stream reduce in the absence of constant production. Therefore, progressive reduction in plasma concentration overtime may reflect reduction in inflammatatory conditions, thus indicating improvement in patient health, for example, in TB [37]. On the other hand, antibodies are more stable, and their levels may continue to remain high for many weeks to months after a patient has recovered. CXCL-10, the most prominently elevated cytokine in COVID-19 patients in this study may be useful as an inflammatory marker related to COVID-19 [38, 39]. In this report, we describe a temporal kinetic analysis of cytokines/chemokines. We speculate that the above alterations in plasma concentration of CXCL-10 are due to the treatment success in these patients indicating resolution of disease. Similar results in the progressive decline of plasma concentrations of CXCL-10 with positive patient outcome has been reported in COVID-19 by others [40], and in TB patients by our group [37]. In contrast, chemokine RANTES levels displayed an upward trend suggesting a protective role of this chemokine in disease progression. RANTES is one of the major chemokines produced by CD8+ T cells in HIV infection to suppress the virus [41]. A recent study showed that RANTES has been elevated in longitudinal samples from subjects with mild COVID-19 [40].

In COVID-19 patients with severe disease (intubated), as compared to those with mild to moderate disease, the median CXCL10 concentration was twice as high. However, this increase was not statistically significant. It is possible that in a larger population of patients the difference may turn out to be significant. Our studies, currently in progress, with enlarged sets of patients in different categories are designed to address this.

Additionally, to facilitate specimen collection and transport our multiplex immunoassay assay works with dried blood spots collected on filter paper tested in several infectious disease models (mouse, monkey, human–unpublished data; these authors) in addition to serum and plasma. Dried blood spots from a finger prick are easily obtained and can be sent through regular mail or courier service even from remote areas to diagnostic test centers.

In light of these, several questions remain to be investigated: what proportion of patients develop antibody responses, post-infection time to seroconversion, differences between patients with short and long incubation periods, and correlation between antibody responses and viral load. Our ongoing studies attempt to address the above issues. The limitations of the current study include the following: 1) only symptomatic infections were enrolled; antibody responses in asymptomatic infection remain to be determined; 2) long-term antibody responses (over several months) were not studied; 3) only a modest patient population was studied. Future studies should explore how the immunity to one coronavirus affects another coronavirus and whether the level of immunity is related to other factors such as age, gender, etc.

### Future directions

This approach can complement the current diagnostic testing platforms that are in use and may show utility in various clinical settings as follows: 1) Antibody responses to vaccination with S protein (in various formats); 2) antibody responses to distinguish between natural infection (N antigen of SARS-CoV-2 inclusion in the multiplex assay is in progress)) and vaccination (S antibodies); 3) ability to identify response to therapy by CXCL-10 measurements; 4) disease severity etc. Further studies are required to support these findings and in light of the complexity of the immunologic profiles noted, the use of predictive analytics tools may also become of value in future studies.

## Supporting information

S1 Fig Titration curve of COVID-19 plasma. The standard curve for SARS-CoV-2 S-RBD antibodies was prepared by two-fold serial dilutions of a plasma sample starting at 1:100 to 1:12,800. The MFI (median fluorescent intensity) values were plotted again the dilution and a 5-parameter logistic (5PL) regression analysis was carried out using GraphPad Prism.
(TIF)

S2 Fig Titers of SARS-CoV-2 IgG antibodies in COVID-19 patients. The interpolated value for each unknown COVID-19 patient sample was obtained by 5PL regression analysis against the standard curve in S1 Fig such that the highest dilution (1/12,800) with the lowest antibody level was set to titer 1, and the lowest was set to 100. Antibody titers in log10 against SARS-CoV-2 in longitudinal samples collected from six COVID-19 patients are shown at estimated time (days) from the onset of symptoms.
(TIF)

S3 Fig The correlation between of SARS-CoV-2 S-RBD IgG titers with MFI (median fluorescent intensity) (R^2^) in longitudinal samples collected from six COVID-19 patients are shown. Antibody titers and MFIs were log transformed.
(TIF)

S1 Table Demographic and clinical information of COVID-19 patients from US. (DOCX)

S2 Table Demographic and clinical information of COVID-19 patients from Pakistan. (DOCX)

S3 Table Antibodies (IgG) against SARS-CoV S protein in rabbits as detected by the multiplex assay. MFI values are shown.
(DOCX)

S4 Table IgG subtypes-(1, 2, 3 and 4) in COVID-19 patients from Pakistan against SARS-CoV-2 S-RBD and SARS-CoV-2 N. MFI values are shown.
(DOCX)

S5 Table Plasma concentrations (pg/mL) of cytokines/chemokines in six COVID-19 patients and healthy from USA as detected by multiplex microbead assay. (DOCX)

S1 Appendix Multiplex data of antibodies (IgG) in COVID-19 patients and healthy individuals from USA. (XLSX)

S2 Appendix Multiplex data of antibodies (IgM) in COVID-19 patients and healthy individuals from USA. (XLSX)

S3 Appendix Multiplex data of antibodies (IgG) in COVID-19 patients and healthy individuals from Pakistan. (XLSX)

S4 Appendix Multiplex data of antibodies (IgG) in disease controls (COPD, TB, RSV and Influenza patients). (XLSX)

S5 Appendix Multiplex data of antibodies (IgG) in COVID-19 and healthy monkeys. (XLSX)

S6 Appendix Multiplex data of antibodies (IgG) in rabbits immunized with SARS-CoV S protein and healthy rabbits. (XLSX)

S7 Appendix Multiplex data of IgG subtypes-1, 2, 3 and 4 in COVID-19 patients from USA. (XLSX)

S8 Appendix Multiplex data of IgG subtypes-1, 2, 3 and 4 in COVID-19 patients from Pakistan. (XLSX)

S9 Appendix COVID-19 patients and healthy individuals from USA- multiplex cytokine detection. (XLSX)

S10 Appendix CXCL-10 (pg/ml) in COVID-19 patients and healthy individuals from Pakistan. (XLSX)

S11 Appendix Assay quantitation- titration curve and titers of SARS-CoV-2 IgG antibodies in COVID-19 patients. (XLSX)

## References

[1] Su S, Wong G, Shi W, Liu J, Lai ACK, Zhou J, et al. Epidemiology, Genetic Recombination, and Pathogenesis of Coronaviruses. Trends Microbiol. 2016;24(6):490–502. doi: 10.1016/j.tim.2016.03.003 27012512 PMC7125511

[2] Weiss SR, Navas-Martin S. Coronavirus pathogenesis and the emerging pathogen severe acute respiratory syndrome coronavirus. Microbiol Mol Biol Rev. 2005;69(4):635–64. doi: 10.1128/MMBR.69.4.635-664.2005 16339739 PMC1306801

[3] Li Q, Guan X, Wu P, Wang X, Zhou L, Tong Y, et al. Early Transmission Dynamics in Wuhan, China, of Novel Coronavirus-Infected Pneumonia. N Engl J Med. 2020;382(13):1199–207. doi: 10.1056/NEJMoa2001316 31995857 PMC7121484

[4] Okba NMA, Muller MA, Li W, Wang C, GeurtsvanKessel CH, Corman VM, et al. Severe Acute Respiratory Syndrome Coronavirus 2-Specific Antibody Responses in Coronavirus Disease Patients. Emerg Infect Dis. 2020;26(7):1478–88. doi: 10.3201/eid2607.200841 32267220 PMC7323511

[5] Ding X. Liu JQL, Fung To S.. Human Coronavirus-229E, -OC43, -NL63, and -HKU1. Reference Module in Life Sciences. 2020;B978-0-12-809633-8.21501-X.

[6] Premkumar L, Segovia-Chumbez B, Jadi R, Martinez DR, Raut R, Markmann A, et al. The receptor binding domain of the viral spike protein is an immunodominant and highly specific target of antibodies in SARS-CoV-2 patients. Sci Immunol. 2020;5(48). doi: 10.1126/sciimmunol.abc8413 32527802 PMC7292505

[7] Lisboa Bastos M, Tavaziva G, Abidi SK, Campbell JR, Haraoui LP, Johnston JC, et al. Diagnostic accuracy of serological tests for covid-19: systematic review and meta-analysis. BMJ. 2020;370:m2516. doi: 10.1136/bmj.m2516 32611558 PMC7327913

[8] Deeks JJ, Raffle AE. Lateral flow tests cannot rule out SARS-CoV-2 infection. BMJ. 2020;371:m4787. doi: 10.1136/bmj.m4787 33310701

[9] Yong SEF, Anderson DE, Wei WE, Pang J, Chia WN, Tan CW, et al. Connecting clusters of COVID-19: an epidemiological and serological investigation. Lancet Infect Dis. 2020;20(7):809–15. doi: 10.1016/S1473-3099(20)30273-5 32330439 PMC7173813

[10] Vetter P, Eckerle I, Kaiser L. Covid-19: a puzzle with many missing pieces. BMJ. 2020;368:m627. doi: 10.1136/bmj.m627 32075791

[11] Arevalo-Rodriguez I, Buitrago-Garcia D, Simancas-Racines D, Zambrano-Achig P, Del Campo R, Ciapponi A, et al. False-negative results of initial RT-PCR assays for COVID-19: A systematic review. PLoS One. 2020;15(12):e0242958. doi: 10.1371/journal.pone.0242958 33301459 PMC7728293

[12] Shete PB, Ravindran R, Chang E, Worodria W, Chaisson LH, Andama A, et al. Evaluation of antibody responses to panels of M. tuberculosis antigens as a screening tool for active tuberculosis in Uganda. PLoS One. 2017;12(8):e0180122. doi: 10.1371/journal.pone.0180122 28767658 PMC5540581

[13] Ravindran R, Krishnan VV, Khanum A, Luciw PA, Khan IH. Exploratory study on plasma immunomodulator and antibody profiles in tuberculosis patients. Clin Vaccine Immunol. 2013;20(8):1283–90. doi: 10.1128/CVI.00213-13 23761664 PMC3754529

[14] Ravindran R, Krishnan VV, Dhawan R, Wunderlich ML, Lerche NW, Flynn JL, et al. Plasma antibody profiles in non-human primate tuberculosis. J Med Primatol. 2014;43(2):59–71. doi: 10.1111/jmp.12097 24446897

[15] Khan IH, Ravindran R, Krishnan VV, Awan IN, Rizvi SK, Saqib MA, et al. Plasma antibody profiles as diagnostic biomarkers for tuberculosis. Clin Vaccine Immunol. 2011;18(12):2148–53. doi: 10.1128/CVI.05304-11 21976221 PMC3232686

[16] Khaliq A, Ravindran R, Hussainy SF, Krishnan VV, Ambreen A, Yusuf NW, et al. Field evaluation of a blood based test for active tuberculosis in endemic settings. PLoS One. 2017;12(4):e0173359. doi: 10.1371/journal.pone.0173359 28380055 PMC5381859

[17] Farr K, Ravindran R, Strnad L, Chang E, Chaisson LH, Yoon C, et al. Diagnostic performance of blood inflammatory markers for tuberculosis screening in people living with HIV. PLoS One. 2018;13(10):e0206119. doi: 10.1371/journal.pone.0206119 30352099 PMC6198956

[18] Dehnad A, Ravindran R, Subbian S, Khan IH. Development of immune-biomarkers of pulmonary tuberculosis in a rabbit model. Tuberculosis (Edinb). 2016;101:1–7. doi: 10.1016/j.tube.2016.07.008 27865378

[19] Ravindran R, Khan IH, Krishnan VV, Ziman M, Kendall LV, Frasier JM, et al. Validation of multiplex microbead immunoassay for simultaneous serodetection of multiple infectious agents in laboratory mouse. J Immunol Methods. 2010;363(1):51–9. doi: 10.1016/j.jim.2010.10.003 20965193

[20] Mani A, Ravindran R, Mannepalli S, Vang D, Luciw PA, Hogarth M, et al. Data mining strategies to improve multiplex microbead immunoassay tolerance in a mouse model of infectious diseases. PLoS One. 2015;10(1):e0116262. doi: 10.1371/journal.pone.0116262 25614982 PMC4304816

[21] Shaan Lakshmanappa Y, Elizaldi SR, Roh JW, Schmidt BA, Carroll TD, Weaver KD, et al. SARS-CoV-2 induces robust germinal center CD4 T follicular helper cell responses in rhesus macaques. Nat Commun. 2021;12(1):541. doi: 10.1038/s41467-020-20642-x 33483492 PMC7822826

[22] Munster VJ, Feldmann F, Williamson BN, van Doremalen N, Pérez-Pérez L, Schulz J, et al. Respiratory disease and virus shedding in rhesus macaques inoculated with SARS-CoV-2. bioRxiv. 2020. doi: 10.1101/2020.03.21.001628 32396922 PMC7486227

[23] Krishnan VV, Ravindran R, Wun T, Luciw PA, Khan IH, Janatpour K. Multiplexed measurements of immunomodulator levels in peripheral blood of healthy subjects: Effects of analytical variables based on anticoagulants, age, and gender. Cytometry B Clin Cytom. 2014;86(6):426–35. doi: 10.1002/cyto.b.21147 24574151

[24] Khan IH, Mendoza S, Yee J, Deane M, Venkateswaran K, Zhou SS, et al. Simultaneous detection of antibodies to six nonhuman-primate viruses by multiplex microbead immunoassay. Clin Vaccine Immunol. 2006;13(1):45–52. doi: 10.1128/CVI.13.1.45-52.2006 16425999 PMC1356626

[25] Jaimes JA, Andre NM, Chappie JS, Millet JK, Whittaker GR. Phylogenetic Analysis and Structural Modeling of SARS-CoV-2 Spike Protein Reveals an Evolutionary Distinct and Proteolytically Sensitive Activation Loop. J Mol Biol. 2020;432(10):3309–25. doi: 10.1016/j.jmb.2020.04.009 32320687 PMC7166309

[26] Dutta NK, Mazumdar K, Gordy JT. The Nucleocapsid Protein of SARS-CoV-2: a Target for Vaccine Development. J Virol. 2020;94(13). doi: 10.1128/JVI.00647-20 32546606 PMC7307180

[27] AN Cohen BK. False positives in reverse transcription PCR testing for SARS-CoV-2. medrxiv. 2020.

[28] Zhu Y YD, Han Y, Yan H, Chong H, Ren L, Wang J, et al. Cross-reactive neutralization of SARS-CoV-2 by serum antibodies from recovered SARS patients and immunized animals. Sci Adv. 2020;2020 Nov; 6(45): eabc9999. doi: 10.1126/sciadv.abc9999 33036961 PMC7673700

[29] Dong S, Sun J, Mao Z, Wang L, Lu YL, Li J. A guideline for homology modeling of the proteins from newly discovered betacoronavirus, 2019 novel coronavirus (2019-nCoV). J Med Virol. 2020;92(9):1542–8. doi: 10.1002/jmv.25768 32181901 PMC7228330

[30] Ng KW, Faulkner N, Cornish GH, Rosa A, Harvey R, Hussain S, et al. Preexisting and de novo humoral immunity to SARS-CoV-2 in humans. Science. 2020;370(6522):1339–43. doi: 10.1126/science.abe1107 33159009 PMC7857411

[31] Zohaib A, Saqib M, Athar MA, Chen J, Sial AU, Khan S, et al. Countrywide Survey for MERS-Coronavirus Antibodies in Dromedaries and Humans in Pakistan. Virol Sin. 2018;33(5):410–7. doi: 10.1007/s12250-018-0051-0 30311100 PMC6235758

[32] Tso FY, Lidenge SJ, Pena PB, Clegg AA, Ngowi JR, Mwaiselage J, et al. High prevalence of pre-existing serological cross-reactivity against SARS-CoV-2 in sub-Sahara Africa. Int J Infect Dis. 2020. 10.1016/j.ijid.2020.10.104 PMC7648883 33176202

[33] Mateus J, Grifoni A, Tarke A, Sidney J, Ramirez SI, Dan JM, et al. Selective and cross-reactive SARS-CoV-2 T cell epitopes in unexposed humans. Science. 2020;370(6512):89–94. doi: 10.1126/science.abd3871 32753554 PMC7574914

[34] Benson DA, Cavanaugh M, Clark K, Karsch-Mizrachi I, Lipman DJ, Ostell J, et al. GenBank. Nucleic Acids Res. 2013;41(Database issue):D36–42. doi: 10.1093/nar/gks1195 23193287 PMC3531190

[35] Amanat F, Stadlbauer D, Strohmeier S, Nguyen THO, Chromikova V, McMahon M, et al. A serological assay to detect SARS-CoV-2 seroconversion in humans. Nat Med. 2020;26(7):1033–6. doi: 10.1038/s41591-020-0913-5 32398876 PMC8183627

[36] Vidarsson G, Dekkers G, Rispens T. IgG subclasses and allotypes: from structure to effector functions. Front Immunol. 2014;5:520. doi: 10.3389/fimmu.2014.00520 25368619 PMC4202688

[37] Chavez K, Ravindran R, Dehnad A, Khan IH. Gender biased immune-biomarkers in active tuberculosis and correlation of their profiles to efficacy of therapy. Tuberculosis (Edinb). 2016;99:17–24. doi: 10.1016/j.tube.2016.03.009 27450000

[38] Zhang N, Zhao YD, Wang XM. CXCL10 an important chemokine associated with cytokine storm in COVID-19 infected patients. Eur Rev Med Pharmacol Sci. 2020;24(13):7497–505. doi: 10.26355/eurrev_202007_21922 32706090

[39] Blot M, Jacquier M, Aho Glele L-S, Beltramo G, Nguyen M, Bonniaud P, et al. CXCL10 could drive longer duration of mechanical ventilation during COVID-19 ARDS. Critical Care. 2020;24(1):632. doi: 10.1186/s13054-020-03328-0 33138839 PMC7604548

[40] Zhao Y, Qin L, Zhang P, Li K, Liang L, Sun J, et al. Longitudinal COVID-19 profiling associates IL-1RA and IL-10 with disease severity and RANTES with mild disease. JCI Insight. 2020;5(13). doi: 10.1172/jci.insight.139834 32501293 PMC7406242

[41] Cocchi F, DeVico AL, Garzino-Demo A, Arya SK, Gallo RC, Lusso P. Identification of RANTES, MIP-1 alpha, and MIP-1 beta as the major HIV-suppressive factors produced by CD8+ T cells. Science. 1995;270(5243):1811–5. doi: 10.1126/science.270.5243.1811 8525373

