# Supplementary figures and images for: Immune response dynamics in COVID-19 patients to SARS-CoV-2 and other human coronaviruses
Source: PLoS One. 2021 Jul 9;16(7):e0254367. doi: 10.1371/journal.pone.0254367 (PMC8270414; doi:10.1371/journal.pone.0254367)

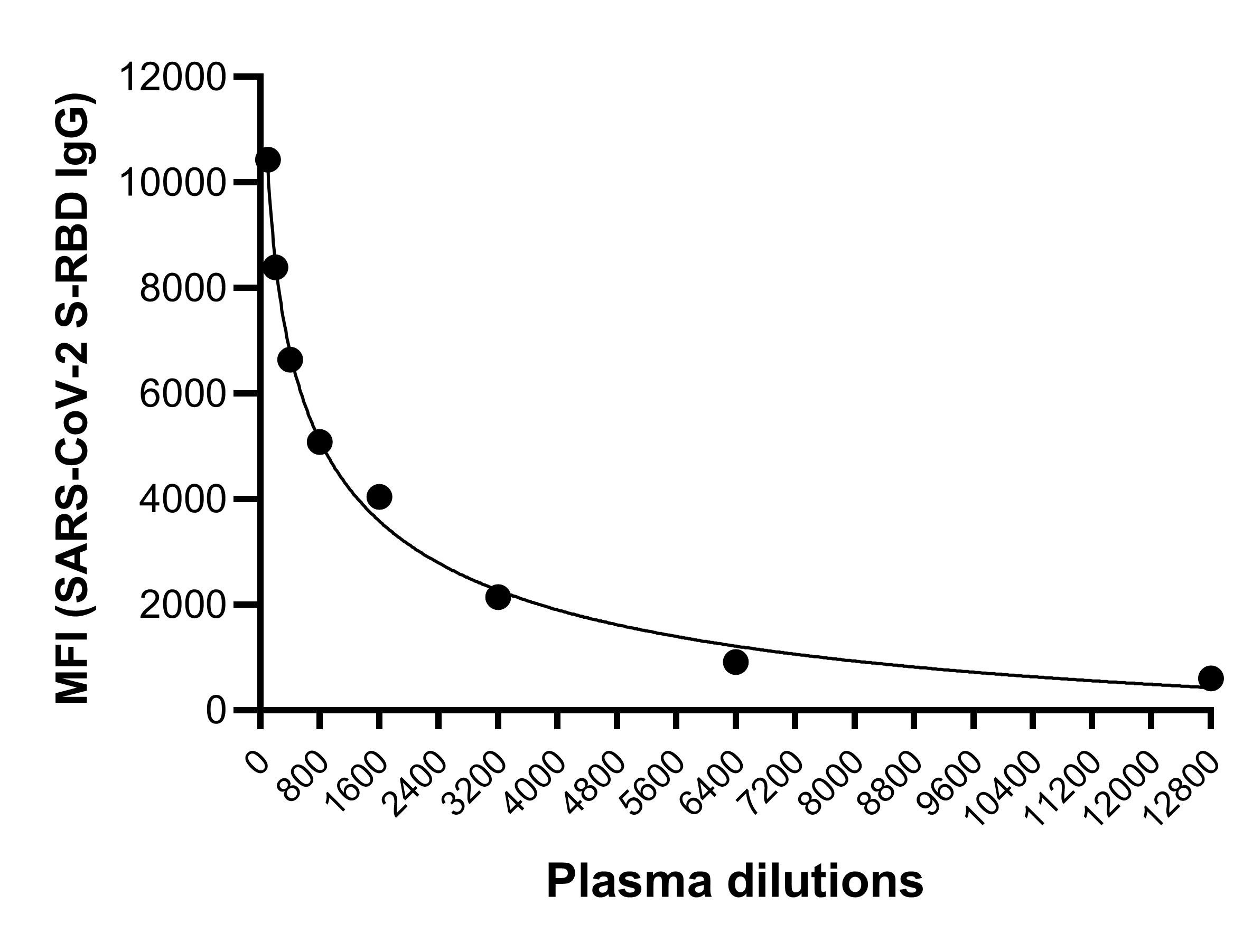

Supplement: S1 Fig — The standard curve for SARS-CoV-2 S-RBD antibodies was prepared by two-fold serial dilutions of a plasma sample starting at 1:100 to 1:12,800. The MFI (median fluorescent intensity) values were plotted again the dilution and a 5-parameter logistic (5PL) regression analysis was carried out using GraphPad Prism. (TIF) [file pone.0254367.s001.tif]

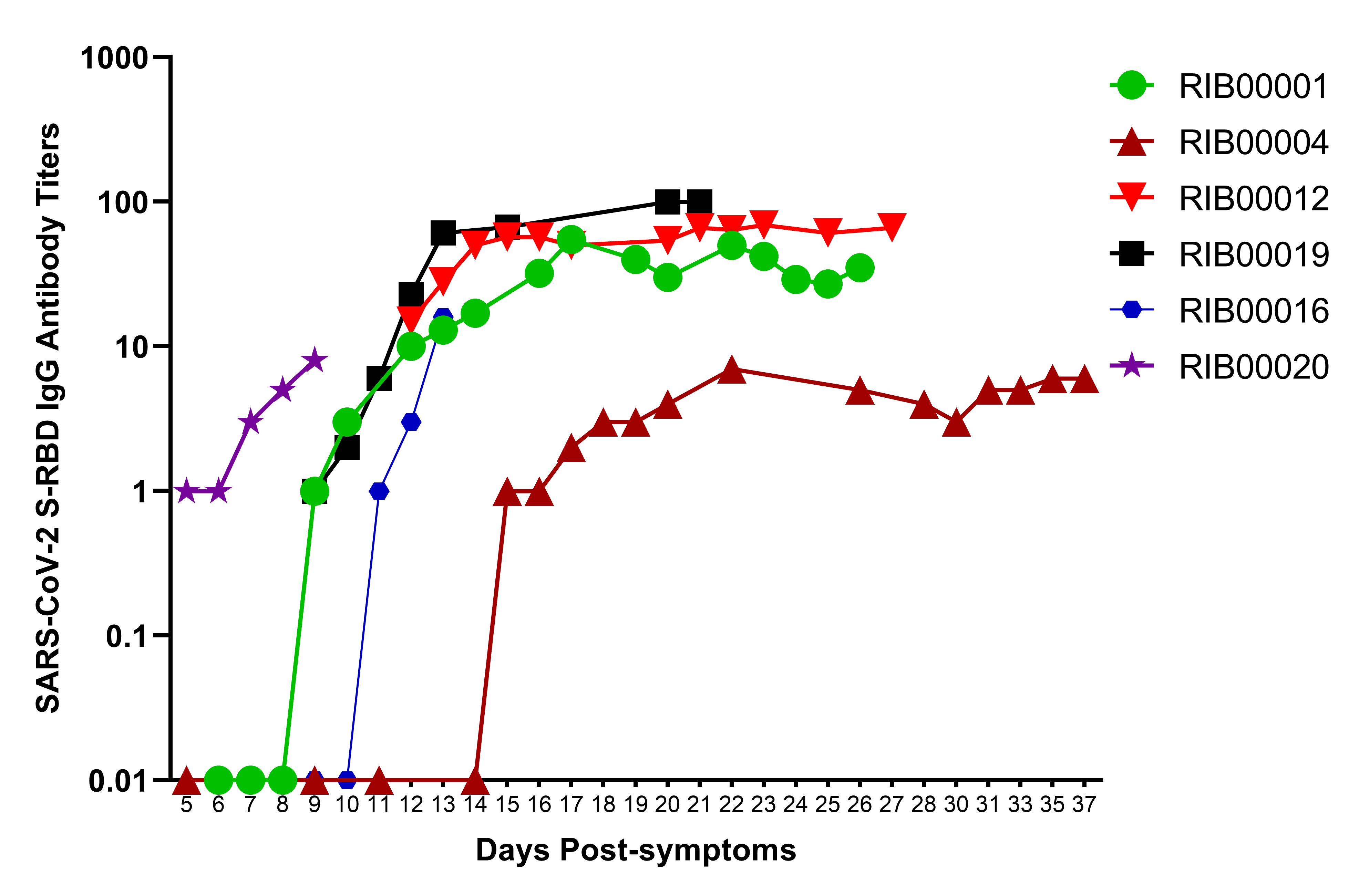

Supplement: S2 Fig — The interpolated value for each unknown COVID-19 patient sample was obtained by 5PL regression analysis against the standard curve in S1 Fig such that the highest dilution (1/12,800) with the lowest antibody level was set to titer 1, and the lowest was set to 100. Antibody titers in log10 against SARS-CoV-2 in longitudinal samples collected from six COVID-19 patients are shown at estimated time (days) from the onset of symptoms. (TIF) [file pone.0254367.s002.tif]

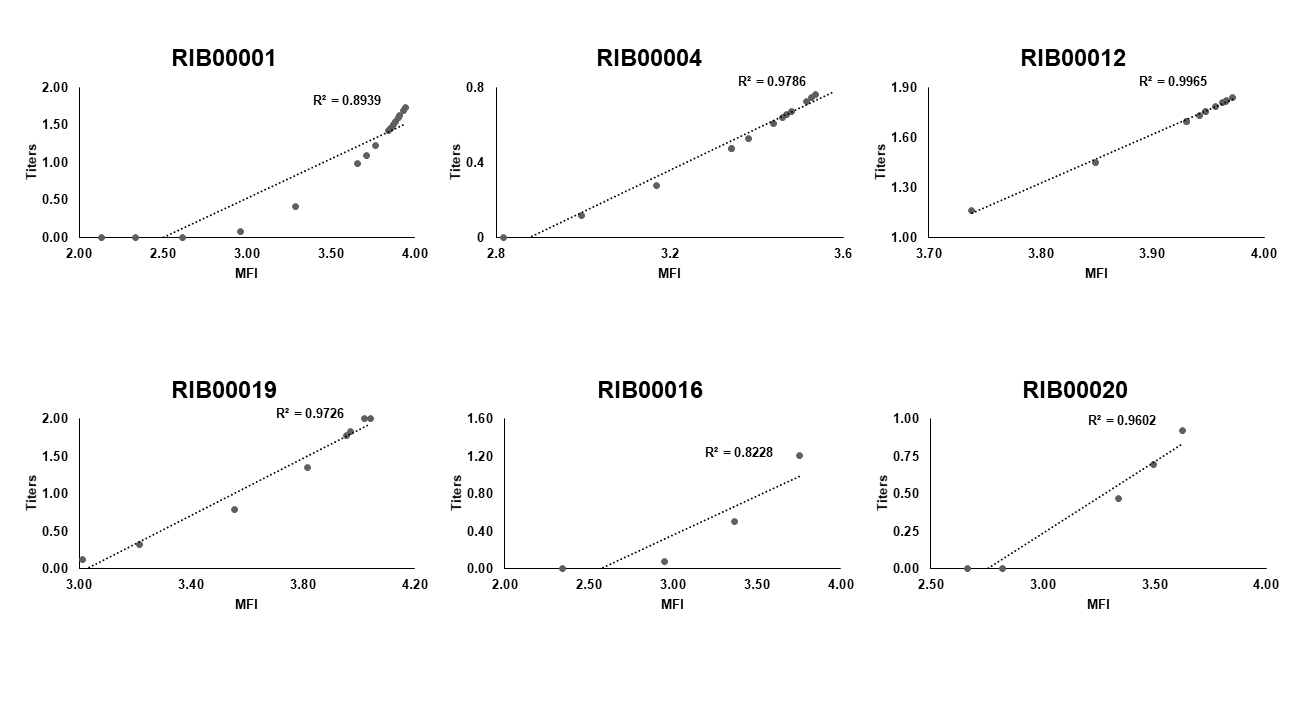

Supplement: S3 Fig — Antibody titers and MFIs were log transformed. (TIF) [file pone.0254367.s003.tif]
