# Supplementary material for: Immune response dynamics in COVID-19 patients to SARS-CoV-2 and other human coronaviruses
Source: PLoS One. 2021 Jul 9;16(7):e0254367. doi: 10.1371/journal.pone.0254367 (PMC8270414; doi:10.1371/journal.pone.0254367)
Supplement: S1 Table — (DOCX) [file pone.0254367.s004.docx]

| **Clinical History of COVID-19 Patients from US** | | | | | | |  |  |  |
| --- | --- | --- | --- | --- | --- | --- | --- | --- | --- |
| **Sample #** | **Gender** | **Age (years)** | **COVID-19 RT-PCR** | **Lengths of Hospital Stay in Days** | **Race** | **COVID-19 Symptoms** | **Onset of COVID-19 Symptoms** | **COVID-19-Comments** | **Airway Procedures Performed** |
| RIB-00001 | Female | 46 | Positive | 26 | Asian | Flu like symptoms, ARDS | Onset of symptoms several days before admission to hospital | Tested positive on day 4 after admission to hospital | Endotracheal Intubation |
| RIB-00004 | Male | 80 | Positive | 54 | Other | Cough, ARDS | 5-8 days before admission to hospital | 1 week prior to admission, admitted for sepsis pneumonia and COVID19 rule out; prolonged intubation/failed intubation also adrenal insufficiency | Endotracheal Intubation |
| RIB-00012 | Male | 71 | Positive | 16 | White | SOB associated with respiratory illness, Cough, Fever, Hypoxemia,  Dyspnea on exertion | 5-8 days before admission to hospital | COVID pos 7 days before admission Required supplemental oxygen on presentation to ED | Supplemental Oxygen |
| RIB-00016 | Male | 61 | Positive | 11 | White | SOB associated with respiratory illness, Cough, AHRF | 9-13 days before admission to hospital | 1 day after admission patient was intubated | Endotracheal Intubation |
| RIB-00020 | Female | 63 | Positive | 4 | Black or African American | SOB associated with respiratory illness, Cough, Flu like symptoms, Dyspnea | 5-8 days before admission to hospital | Positive Covid test | No invasive procedures performed |
| RIB-00019 | Male | 51 | Positive | 13 | Black or African American | SOB associated with respiratory illness, Cough, Flu like symptoms, Chest pain, Fever | 9-13 days before admission to hospital | Supplemental oxygen on presentation | No invasive procedures performed |
| **Abbreviations: SOB (Shortness of breath); ARDS (Acute Respiratory Distress Syndrome); (AHRF) Acute Hypoxic Respiratory Failure** | | | | | | | | | |
