# Supplementary material for: Immune response dynamics in COVID-19 patients to SARS-CoV-2 and other human coronaviruses
Source: PLoS One. 2021 Jul 9;16(7):e0254367. doi: 10.1371/journal.pone.0254367 (PMC8270414; doi:10.1371/journal.pone.0254367)
Supplement: S2 Table — (DOCX) [file pone.0254367.s005.docx]

| **Clinical History of COVID-19 Patients from Pakistan** | | | | | | |  |  |  |
| --- | --- | --- | --- | --- | --- | --- | --- | --- | --- |
| **Sample #** | **Gender** | **Age (Years)** | **COVID-19 RT-PCR** | **Lengths of Hospital Stay in Days** | **Race** | **COVID-19 Symptoms** | **Onset of COVID-19 Symptoms** | **COVID-19-Comments** | **Airway Procedures Performed** |
| **Severe COVID-19 Patients, n=20** |  |  |  |  |  |  |  |  |  |
| Covid-3/20 | Male | 58 | Positive | 17 | Asian | Moderate Headache | N/A | 16 days in ICU | No invasive procedures performed |
| Covid-6/20 | Male | 65 | Positive | 1 | Asian | Mild SOB, Moderate cough | N/A | 2 days in ICU | Endotracheal Intubation |
| Covid-10/20 | Male | 52 | Positive | not available | Asian | ARDS, Severe SOB, Mild chest pain | N/A | Not in ICU | No invasive procedures performed |
| Covid-11/20 | Male | 41 | Positive | 12 | Asian | ARDS, Mild SOB, , Moderate cough | N/A | 4 days in ICU | No invasive procedures performed |
| Covid-12/20 | Female | 70 | Positive | not available | Asian | ARDS, Mild SOB, Mild headache, Chills, , Mild cough | N/A | 30 days in ICU | No invasive procedures performed |
| Covid-14/20 | Female | 65 | Positive | 1 | Asian | Moderate SOB | N/A | 1 day in ICU | No invasive procedures performed |
| Covid-15/20 | Female | 40 | Positive | 9 | Asian | Mild SOB, Moderate headache, , Mild cough | N/A | 8 days in ICU | No invasive procedures performed |
| Covid-16/20 | Male | 68 | Positive | 16 | Asian | Moderate SOB, Chills, Mild Chest Pain, , Moderate cough | N/A | 4 days in ICU | Endotracheal Intubation |
| Covid-17/20 | Male | 67 | Positive | 14 | Asian | ARDS, Severe SOB, , Mild cough | N/A | 3 days in ICU | No invasive procedures performed |
| Covid-18/20 | Male | 77 | Positive | 12 | Asian | ARDS, Severe SOB, , Mild cough | N/A | 12 days in ICU | Endotracheal Intubation |
| Covid-20/20 | Female | 70 | Positive | 6 | Asian | ARDS, Moderate SOB, Mild headache, Chills, , Moderate cough | N/A | 2 days in ICU | No invasive procedures performed |
| Covid-21/20 | Male | 43 | Positive | 25 | Asian | Mild SOB, Chills, , Mild cough | N/A | 11 days in ICU | No invasive procedures performed |
| Covid-22/20 | Male | 70 | Positive | 10 | Asian | Severe SOB, Mild headache, Chills, , Severe cough | N/A | 3 days in ICU | No invasive procedures performed |
| Covid-26/20 | Male | 73 | Positive | 10 | Asian | ARDS, Severe SOB, Moderate chest pain | N/A | 10 days in ICU | Endotracheal Intubation |
| Covid-29/20 | Male | 53 | Positive | 13 | Asian | ARDS, Severe SOB | N/A | 13 days in ICU | Endotracheal Intubation |
| Covid-32/20 | Female | 82 | Positive | 1 | Asian | Mild SOB, Chills | N/A | Not in ICU | No invasive procedures performed |
| Covid-44/20 | Male | 70 | Positive | 19 | Asian | ARDS, Moderate SOB, Mild Chest Pain | N/A | 1 day in ICU | No invasive procedures performed |
| Covid-48/20 | Female | 55 | Positive | 2 | Asian | ARDS, Mild SOB, , Mild cough | N/A | Not in ICU | No invasive procedures performed |
| Covid-49/20 | Male | 55 | Positive | 12 | Asian | Mild SOB, Mild headache, Moderate chest pain, , Mild cough | N/A | Not in ICU | No invasive procedures performed |
| Covid-50/20 | Male | 43 | Positive | 9 | Asian | Mild headache, Chills, Mild chest pain, , Severe cough | N/A | 1 day in ICU | No invasive procedures performed |
| **Mild/moderate COVID-19 Patients, n=23** |  |  |  |  |  |  |  |  |  |
| Covid-1/20 | Male | 59 | Positive | 12 | Asian | Mild SOB, Mild headache, , Moderate cough | N/A | Not in ICU | No invasive procedures performed |
| Covid-2/20 | Male | 80 | Positive | 3 | Asian | Severe SOB | N/A | Not in ICU | No invasive procedures performed |
| Covid-4/20 | Female | 65 | Positive | 5 | Asian | Mild SOB, Mild headache, Mild cough | N/A | Not in ICU | No invasive procedures performed |
| Covid-5/20 | Male | 45 | Positive | 4 | Asian | Mild SOB, Mild Chest Pain , Mild cough | N/A | Not in ICU | No invasive procedures performed |
| Covid-7/20 | Female | 69 | Positive | 24 | Asian | , Mild cough | N/A | Not in ICU | No invasive procedures performed |
| Covid-8/20 | Male | 31 | Positive | 3 | Asian | Mild SOB, Mild cough | N/A | Not in ICU | No invasive procedures performed |
| Covid-19/20 | Male | 81 | Positive | 8 | Asian | not available | N/A | Not in ICU | No invasive procedures performed |
| Covid-23/20 | Male | 58 | Positive | 7 | Asian | Severe SOB, Headache, Chills , Moderate cough | N/A | Not in ICU | No invasive procedures performed |
| Covid-24/20 | Female | 60 | Positive | not available | Asian | ARDS, Moderate SOB, Mild Chest Pain , Moderate cough | N/A | Not in ICU | No invasive procedures performed |
| Covid-25/20 | Male | 65 | Positive | 11 | Asian | Mild SOB, Mild Headache, Chills, Mild cough | N/A | Not in ICU | No invasive procedures performed |
| Covid-27/20 | Male | 59 | Positive | 4 | Asian | Mild SOB, Chills , Mild cough | N/A | Not in ICU | No invasive procedures performed |
| Covid-28/20 | Male | 60 | Positive | 8 | Asian | Mild SOB, Mild cough | N/A | Not in ICU | No invasive procedures performed |
| Covid-31/20 | Male | 55 | Positive | 3 | Asian | Moderate SOB, Mild Chest Pain , Mild cough | N/A | Not in ICU | Endotracheal Intubation |
| Covid-33/20 | Female | 60 | Positive | 30 | Asian | Severe SOB, Chills, Severe cough | N/A | Not in ICU | No invasive procedures performed |
| Covid-34/20 | Male | 50 | Positive | 3 | Asian | Severe Chest pain , Severe cough | N/A | Not in ICU | No invasive procedures performed |
| Covid-35/20 | Male | 58 | Positive | 5 | Asian | Severe SOB, Severe headache | N/A | Not in ICU | No invasive procedures performed |
| Covid-36/20 | Male | 57 | Positive | 18 | Asian | Severe SOB, Chills, Mild chest pain | N/A | Not in ICU | No invasive procedures performed |
| Covid-37/20 | Male | 51 | Positive | 12 | Asian | Moderate SOB | N/A | Not in ICU | No invasive procedures performed |
| Covid-38/20 | Male | 51 | Positive | 5 | Asian | Mild SOB, Severe chest pain , Severe cough | N/A | Not in ICU | No invasive procedures performed |
| Covid-39/20 | Female | 28 | Positive | 8 | Asian | Mild SOB, Mild headache, Mild chest pain , Mild cough | N/A | Not in ICU | No invasive procedures performed |
| Covid-41/20 | Male | 47 | Positive | 4 | Asian | Severe SOB, Chills | N/A | Not in ICU | No invasive procedures performed |
| Covid-42/20 | Female | 57 | Positive | 3 | Asian | Severe SOB, Mild Headache | N/A | Not in ICU | No invasive procedures performed |
| Covid-45/20 | Male | 75 | Positive | 20 | Asian | Severe SOB | N/A | Not in ICU | No invasive procedures performed |
| **Abbreviations: SOB (Shortness of breath); ARDS (Acute Respiratory Distress Syndrome); N/A (not available)** | | | | | | | | | |
