# Supplementary material for: Immune response dynamics in COVID-19 patients to SARS-CoV-2 and other human coronaviruses
Source: PLoS One. 2021 Jul 9;16(7):e0254367. doi: 10.1371/journal.pone.0254367 (PMC8270414; doi:10.1371/journal.pone.0254367)
Supplement: S3 Table — MFI values are shown. (DOCX) [file pone.0254367.s006.docx]

| **Rabbits-Multiplex antibody (IgG) detection (Median Fluorescence Intensities)** | |
| --- | --- |
| **Sample #** | **SARS-CoV S** |
| **Healthy Rabbits, N=9** | |
| 829 | 186 |
| 821 | 94 |
| 853 | 109 |
| 820 | 101 |
| 833 | 104 |
| 857 | 150 |
| 863 | 80 |
| NRC-769 | 130 |
| NRC-774 | 81 |
| **Rabbits immunised with SARS-CoV S protein, N=6** | |
| NRC-770 | 18027 |
| NRC-771 | 20802 |
| NRC-772 | 21372 |
| NRC-775 | 20837 |
| NRC-776 | 21360 |
| NRC-777 | 21542 |
