# Supplementary material for: Immune response dynamics in COVID-19 patients to SARS-CoV-2 and other human coronaviruses
Source: PLoS One. 2021 Jul 9;16(7):e0254367. doi: 10.1371/journal.pone.0254367 (PMC8270414; doi:10.1371/journal.pone.0254367)
Supplement: S4 Table — MFI values are shown. (DOCX) [file pone.0254367.s007.docx]

| **IgG subtypes-1, 2, 3 and 4 in COVID-19 patients from Pakistan (Median Fluorescence Intensities)** | | | | | | |  |  |
| --- | --- | --- | --- | --- | --- | --- | --- | --- |
|  | **SARS-CoV-2 S-RBD** | | | | **SARS-CoV N** | | | |
| Sample# | **IgG1** | **IgG2** | **IgG3** | **IgG4** | **IgG1** | **IgG2** | **IgG3** | **IgG4** |
| **Severe COVID-19, N=20** | | | | | | | | |
| Covid Pak-3/20 | 24 | 23 | 24 | 38 | 19 | 22 | 20 | 39 |
| Covid Pak-6/20 | 28 | 24 | 24 | 39 | 25 | 22 | 20 | 33 |
| Covid Pak-10/20 | 13214 | 56 | 83 | 38 | 2434 | 23 | 545 | 39 |
| Covid Pak-11/20 | 2069 | 27 | 37 | 37 | 194 | 70 | 21 | 38 |
| Covid Pak-12/20 | 24 | 26 | 52 | 39 | 16 | 20 | 91 | 37 |
| Covid Pak-14/20 | 23 | 24 | 24 | 38 | 26 | 23 | 19 | 38 |
| Covid Pak-15/20 | 848 | 28 | 211 | 36 | 458 | 21 | 441 | 33 |
| Covid Pak-16/20 | 944 | 191 | 78 | 36 | 1020 | 47 | 3921 | 35 |
| Covid Pak-17/20 | 142 | 91 | 440 | 36 | 585 | 1662 | 9643 | 52 |
| Covid Pak-18/20 | 7940 | 35 | 486 | 36 | 10144 | 222 | 1148 | 44 |
| Covid Pak-20/20 | 2036 | 35 | 205 | 35 | 431 | 21 | 103 | 36 |
| Covid Pak-21/20 | 7673 | 38 | 61 | 38 | 11154 | 286 | 250 | 48 |
| Covid Pak-22/20 | 13768 | 102 | 331 | 57 | 16334 | 154 | 1164 | 75 |
| Covid Pak-26/20 | 9025 | 107 | 339 | 37 | 1872 | 109 | 2070 | 208 |
| Covid Pak-29/20 | 2492 | 30 | 66 | 40 | 872 | 293 | 181 | 1438 |
| Covid Pak-32/20 | 9728 | 42 | 413 | 35 | 10088 | 335 | 1011 | 39 |
| Covid Pak-44/20 | 22 | 27 | 94 | 35 | 215 | 201 | 12745 | 35 |
| Covid Pak-48/20 | 188 | 27 | 65 | 34 | 90 | 20 | 37 | 31 |
| Covid Pak-49/20 | 1701 | 204 | 390 | 72 | 1657 | 504 | 524 | 87 |
| Covid Pak-50/20 | 35 | 72 | 192 | 76 | 414 | 203 | 16119 | 78 |
| **Mild/moderate COVID-19, N=23** | | | | | | | | |
| Covid Pak-1/20 | 565 | 25 | 132 | 34 | 1921 | 26 | 4119 | 33 |
| Covid Pak-2/20 | 24 | 26 | 28 | 36 | 59 | 35 | 520 | 34 |
| Covid Pak-4/20 | 22 | 23 | 23 | 37 | 17 | 22 | 19 | 35 |
| Covid Pak-5/20 | 631 | 26 | 34 | 33 | 81 | 24 | 25 | 35 |
| Covid Pak-7/20 | 3883 | 40 | 71 | 36 | 7825 | 110 | 557 | 41 |
| Covid Pak-8/20 | 1216 | 27 | 28 | 34 | 71 | 23 | 20 | 35 |
| Covid Pak-19/20 | 1015 | 29 | 90 | 36 | 11183 | 491 | 184 | 40 |
| Covid Pak-23/20 | 607 | 25 | 41 | 37 | 723 | 21 | 49 | 35 |
| Covid Pak-24/20 | 25 | 27 | 68 | 38 | 207 | 5980 | 68 | 37 |
| Covid Pak-25/20 | 38 | 68 | 317 | 35 | 241 | 153 | 1247 | 38 |
| Covid Pak-27/20 | 44 | 42 | 253 | 33 | 76 | 74 | 448 | 40 |
| Covid Pak-28/20 | 472 | 27 | 38 | 36 | 9906 | 409 | 133 | 50 |
| Covid Pak-31/20 | 36 | 29 | 28 | 37 | 1859 | 29 | 33 | 42 |
| Covid Pak-33/20 | 34 | 28 | 89 | 35 | 473 | 119 | 12769 | 40 |
| Covid Pak-34/20 | 1152 | 29 | 40 | 35 | 936 | 21 | 102 | 30 |
| Covid Pak-35/20 | 5845 | 29 | 54 | 34 | 2088 | 39 | 13181 | 33 |
| Covid Pak-36/20 | 3302 | 36 | 140 | 38 | 3730 | 5437 | 5019 | 36 |
| Covid Pak-37/20 | 27 | 25 | 93 | 31 | 98 | 43 | 11866 | 33 |
| Covid Pak-38/20 | 3869 | 28 | 59 | 35 | 896 | 20 | 50 | 34 |
| Covid Pak-39/20 | 3922 | 39 | 226 | 36 | 12060 | 40 | 509 | 33 |
| Covid Pak-41/20 | 48 | 755 | 35 | 36 | 17 | 18 | 56 | 30 |
| Covid Pak-42/20 | 29 | 22 | 34 | 32 | 29 | 19 | 42 | 34 |
| Covid Pak-45/20 | 2206 | 26 | 24 | 35 | 39 | 21 | 26 | 34 |
