# Supplementary material for: Immune response dynamics in COVID-19 patients to SARS-CoV-2 and other human coronaviruses
Source: PLoS One. 2021 Jul 9;16(7):e0254367. doi: 10.1371/journal.pone.0254367 (PMC8270414; doi:10.1371/journal.pone.0254367)
Supplement: S5 Table — (DOCX) [file pone.0254367.s008.docx]

| **COVID-19 patients and healthy individuals from USA-multiplex cytokine detection (pg/mL)** | | | | | | | |  |  |
| --- | --- | --- | --- | --- | --- | --- | --- | --- | --- |
| **Sample#** | **Approximate days post-symptoms** | **CXCL-10** | **MCP-1** | **IL-2R aplha** | **Eotaxin** | **G-CSF** | **GRO-a** | **SCGF-b** | **RANTES** |
| **COVID-19 UCDavis: Time course N=6** | | |  |  |  |  |  |  |  |
| RIB-00001-LH-02 | 6 | 127320 | 219 | 720 | 121 | 549 | 1150 | 545041 | 17340 |
| RIB-00001-LH-03 | 7 | 127320 | 247 | 653 | 115 | 514 | 1413 | 343717 | 27607 |
| RIB-00001-LH-04 | 8 | 127320 | 669 | 753 | 146 | 896 | 585 | 464745 | 14297 |
| RIB-00001-LH-05 | 9 | 67863 | 281 | 717 | 102 | 570 | 806 | 477578 | 27440 |
| RIB-00001-LH-06 | 10 | 12320 | 137 | 457 | 73 | 296 | 620 | 356358 | 13176 |
| RIB-00004-LH-09 | 14 | 3520 | 90 | 138 | 153 | 286 | 744 | 728527 | 13579 |
| RIB-00004-LH-10 | 15 | 2359 | 41 | 179 | 222 | 178 | 943 | 821271 | 12214 |
| RIB-00004-LH-11 | 16 | 1866 | 49 | 219 | 214 | 225 | 1120 | 794165 | 26635 |
| RIB-00004-LH-12 | 17 | 1317 | 50 | 181 | 141 | 183 | 998 | 689030 | 14709 |
| RIB-00004-LH-13 | 18 | 1161 | 27 | 221 | 131 | 253 | 1219 | 675924 | 40658 |
| RIB-00012-LH-0 | 12 | 6860 | 435 | 239 | 348 | 441 | 927 | 575763 | 303629 |
| RIB-00012-LH-01 | 13 | 4585 | 477 | 209 | 301 | 393 | 1009 | 574953 | 303629 |
| RIB-00012-LH-02 | 14 | 3559 | 353 | 228 | 250 | 476 | 695 | 472706 | 303629 |
| RIB-00012-LH-03 | 15 | 1195 | 203 | 151 | 171 | 239 | 659 | 532437 | 303629 |
| RIB-00012-LH-04 | 16 | 928 | 210 | 288 | 270 | 162 | 658 | 563721 | 395663 |
| RIB-00016-LH-0 | 9 | 5430 | 230 | 132 | 190 | 166 | 849 | 466980 | 15990 |
| RIB-00016-LH-01 | 10 | 5698 | 470 | 75 | 209 | 178 | 954 | 393475 | 59399 |
| RIB-00016-LH-02 | 11 | 6752 | 432 | 65 | 157 | 211 | 1037 | 473402 | 21164 |
| RIB-00016-LH-03 | 12 | 5941 | 497 | 79 | 183 | 175 | 1014 | 614635 | 19283 |
| RIB-00016-LH-04 | 13 | 2326 | 161 | 84 | 112 | 119 | 976 | 531077 | 86959 |
| RIB-00019-LH-0 | 9 | 3430 | 40 | 72 | 30 | 110 | 1318 | 258550 | 47538 |
| RIB-00019-LH-01 | 10 | 2578 | 40 | 55 | 32 | 69 | 967 | 289154 | 69263 |
| RIB-00019-LH-02 | 11 | 1737 | 32 | 44 | 36 | 107 | 1139 | 283073 | 395663 |
| RIB-00019-LH-03 | 12 | 2000 | 45 | 30 | 23 | 23 | 792 | 190656 | 20593 |
| RIB-00019-LH-04 | 13 | 1416 | 21 | 20 | 27 | 54 | 892 | 180111 | 208100 |
| RIB-00020-LH-0 | 5 | 3292 | 102 | 184 | 135 | 86 | 785 | 309390 | 10302 |
| RIB-00020-LH-01 | 6 | 3224 | 87 | 173 | 104 | 152 | 1000 | 316894 | 31473 |
| RIB-00020-LH-02 | 7 | 1708 | 120 | 206 | 161 | 70 | 919 | 352491 | 12148 |
| RIB-00020-LH-03 | 8 | 677 | 101 | 158 | 155 | 76 | 859 | 248243 | 15048 |
| RIB-00020-LH-04 | 9 | 339 | 78 | 136 | 121 | 64 | 621 | 197974 | 28286 |
| **Healthy, N=16** |  |  |  |  |  |  |  |  |  |
| 001H |  | 306 | 34 | 59 | 58 | 14 | 567 | 47905 | 3329 |
| 002H |  | 595 | 47 | 81 | 184 | 16 | 451 | 49857 | 4230 |
| 003H |  | 354 | 80 | 79 | 104 | 14 | 621 | 71190 | 5822 |
| 004H |  | 260 | 62 | 65 | 112 | 31 | 779 | 59238 | 3076 |
| 005H |  | 258 | 65 | 70 | 78 | 45 | 1078 | 113889 | 5373 |
| 006H |  | 227 | 39 | 86 | 134 | 27 | 6 | 70265 | 2580 |
| 007H |  | 135 | 25 | 136 | 65 | 40 | 1159 | 95508 | 43656 |
| 008H |  | 323 | 32 | 72 | 105 | 54 | 905 | 59390 | 7452 |
| 009H |  | 238 | 43 | 95 | 127 | 27 | 765 | 65041 | 3522 |
| 010H |  | 206 | 30 | 70 | 100 | 36 | 922 | 42381 | 8485 |
| 011H |  | 103 | 27 | 68 | 62 | 40 | 908 | 27434 | 2913 |
| 012H |  | 1880 | 217 | 93 | 113 | 111 | 1133 | 94564 | 3850 |
| 013H |  | 157 | 23 | 68 | 58 | 10 | 1010 | 33825 | 5200 |
| 014H |  | 506 | 26 | 59 | 71 | 14 | 6 | 82350 | 1531 |
| 015H |  | 135 | 43 | 61 | 86 | 14 | 1107 | 84065 | 6358 |
| 016H |  | 358 | 36 | 56 | 116 | 22 | 728 | 51362 | 2802 |
